# Designed heterogeneous palladium catalysts for reversible light-controlled bioorthogonal catalysis in living cells

**DOI:** 10.1038/s41467-018-03617-x

**Published:** 2018-03-23

**Authors:** Faming Wang, Yan Zhang, Zhi Du, Jinsong Ren, Xiaogang Qu

**Affiliations:** 1 Laboratory of Chemical Biology and State Key Laboratory of Rare Earth Resource Utilization, Changchun Institute of Applied Chemistry, Chinese Academy of Sciences, Changchun, 130022 Jilin China; 2 University of Chinese Academy of Sciences, Beijing, 100039 China

## Abstract

As a powerful tool for chemical biology, bioorthogonal chemistry broadens the ways to explore the mystery of life. In this field, transition metal catalysts (TMCs) have received much attention because TMCs can rapidly catalyze chemical transformations that cannot be accomplished by bio-enzymes. However, fine controlling chemical reactions in living systems like bio-enzymes is still a great challenge. Herein, we construct a versatile light-controlled bioorthogonal catalyst by modifying macroporous silica-Pd^0^ with supramolecular complex of azobenzene (Azo) and β-cyclodextrin (CD). Its catalytic activity can be regulated by light-induced structural changes, mimicking allosteric regulation mechanism of bio-enzymes. The light-gated heterogeneous TMCs are important for in situ controlling bioorthogonal reactions and have been successfully used to synthesize a fluorescent probe for cell imaging and mitochondria-specific targeting agent by Suzuki–Miyaura cross-coupling reaction. Endowing the bioorthogonal catalyst with new functions is highly valuable for realizing more complex researches in biochemistry.

## Abstract (web summary)

Fine controlling chemical reactions over transition metal catalysts in living systems like enzymes remains a challenge. Here, the authors develop a versatile light-controlled bioorthogonal catalyst by modifying macroporous silica-Pd^0^ with supramolecular complex of azobenzene and β-cyclodextrin.

## Introduction

Bioorthogonal chemistry has attracted much attention and played critical important roles to dissect complex intracellular processes and perform specific chemical reactions in living systems^1–3^. Being a relatively new sector of chemical biology, bioorthogonal chemistry has made significant progress in imaging, regulation, and therapeutic applications^4–6^. Different kinds of bioorthogonal reactions have been gratifyingly realized in living systems^7,8^. Transition metal catalysts (TMCs) become outstanding candidates for catalyzing different bioorthogonal reactions^9–12^. Compared with natural enzyme systems, TMCs can rapidly catalyze chemical transformations that cannot be realized by natural enzymes for specific biological applications^13–15^. However, in terms of accuracy and intelligence, current catalytic behaviors of TMCs are undeniably far less than natural biological processes. In fact, life is a highly ordered system and all the activities of proteins, genes, and other signal molecules are precisely controlled in timing and location. Molecules arranged in a random fashion cannot form a living system^16,17^. That means, reactions in living systems are intelligently controlled on demand. Therefore, for practical applications, it is highly desirable and demanding to precisely determine when, where, and to what extent a bioorthogonal reaction is started and ended. Unfortunately, most of previously reported bioorthogonal catalysts do not provide the capability of precisely controlling the catalytic processes according to extrinsic stimulus.

To this end, people developed different extrinsic stimulus to control bioorthogonal reactions by liberating reactive groups through mild chemistry or light^18–20^. Though it has made significant advances in responsive bioorthogonal reactions, these strategies rely on design of precursors or photoremovable protecting groups (PPGs), limiting the reaction types in Cu-click or photo-click reactions^21–24^. Moreover, the extra chemical reactions would increase the complexity and indeterminacy inevitably. The leave of PPGs usually needs high extra energy along with relatively slow reaction rates, and the PPGs have irreversibility and instability under certain conditions. Recently, cucurbit[7]uril has been used to block the access to the catalytic site of Ru or Pd catalysts on the surface of Au nanoparticles, and the catalyst can be re-activated by extra addition of 1-adamantylamine (ADA)^25^. This is an innovative design for TMC-mediated bioorthogonal chemistry. However, extra addition may cause potential interference in cellular processes and is still difficult to realize reversible control and temporal-spatial resolution.

**Fig. 1:**
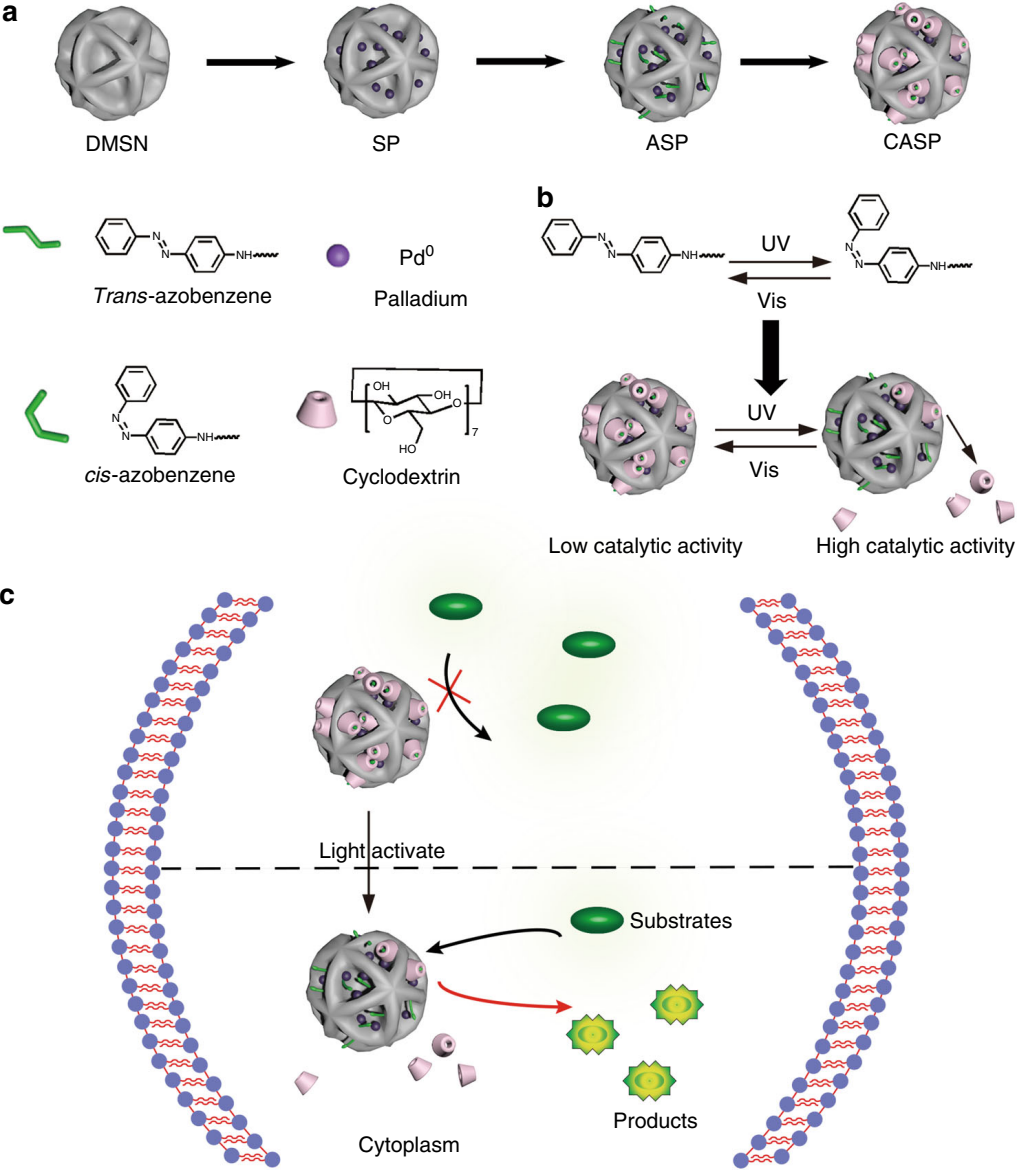
Illustration of design of photo-responsive bioorthogonal catalysts in cells. **a** Macroporous silica (DMSN), Pd-embedded silica nanoparticles (SP), Azo-modified complex catalyst (ASP), and CD-capped complex catalyst (CASP) used in the study. **b** The photo-isomerization process between *trans*-azobenzene and *cis*-azobenzene can cause the dissociation and recombination of CD. **c** Intracellular bioorthogonal catalysis with CASP

Light is an ideal external trigger for manipulating chemical reactions^26^. It is highly selective and also harmless when applied properly. Several excellent examples have successfully applied light in living systems with temporal-spatial resolution^27–32^. Meggers and co-workers reported that the organometallic complex [Cp*Ru(η^6^-pyrene)]PF_6_ (Cp* = pentamethylcyclopentadienyl) could catalyze the conversion of *N*-allylcarbamates into their amines in the presence of thiols, water, and air under UV light^33^. However, the homogeneous organometallic catalyst [Cp*Ru(η^6^-pyrene)]PF_6_ was difficult in synthesis, separation, and purification. Moreover, the application of the homogeneous organometallic catalysts in living cells was challenging due to the issues of biocompatibility, water solubility, stability, and rapid efflux of catalysts from living cells^10,25^. Currently, although many TMCs have been used to catalyze multiple bioorthogonal reactions, light-controllable heterogeneous TMCs have not been reported. Herein, we designed Pd nanoparticles-imbedded macroporous silica nanoparticles (SP), then modified the nanocatalyst with an azobenzene switch, abbreviated as ASP (Fig. 1)^34^. The resulting catalyst can be reversibly functionalized by cyclodextrin (CD) through host–guest interactions and named as CASP. When the access to catalytic site of CASP is blocked by CD, the catalytic activity will be inhibited. By utilizing the isomerization of Azo, the CD blocker can be released from the catalysts resulting in the recovery of catalytic activity under UV illumination or returned to the original inactive status again under visible light^35^. Since the energy band between isomerization of Azo is much lower than the bond energy of PPGs^36^, lower dose of extra light is needed. The efficacy of our designed heterogeneous catalyst was verified in cuvettes and living cells through two applications: (1) the gated generation of fluorophore through deallylation of non-fluorescent precursors;^9,37^ (2) the gated synthesis of a mitochondria-specific probe in situ by Suzuki–Miyaura cross-coupling reaction and light-controllable activation of a prodrug in living cells^10^. Herein, there are three major advantages of our designed catalysts: (1) the synthetic method of the heterogeneous TMCs is versatile and easy to be applied for constructing different kinds of catalysts, such as Pt, Ru, Rh, and so on. (2) The reversible interaction between Azo and CD endows the bioorthogonal catalyst with well reversibility, providing a great promise for broadening potential application of bioorthogonal catalysts in biosystem. (3) Low-dosed light as the external stimulus is an accepted green regulation method and possesses excellent controllability in space and time.

In this study, we demonstrate that the designed heterogeneous catalyst could effectively mediate the bioorthogonal reactions in situ through light. Such light-gated control of bioorthogonal catalysis in living cells has not been demonstrated to date although this issue is important for activating catalytic process at target locations and artificially changing catalytic activity to maintain homeostasis for long-term therapeutics.

## Results

### Design and construction of the complex nanocatalyst

Encouraged by the vast possibilities and applications of palladium chemistry, the preparation of a bio-friendly light-controlled Pd^0^-based heterogeneous catalyst was undertaken. The detailed synthesis process was provided in supporting information. Briefly, macroporous silica nanoparticles (DMSN) were used as the scaffold to stabilize the transition metal (Pd^0^)^38^. The average particle diameter and central-radial pore size of DMSN were 80 nm and 12.3 nm, respectively, as indicated by scanning electron microscopy (SEM), transmission electron microcopy (TEM) images (Supplementary Fig. 1a, b), and nitrogen adsorption measurements (Supplementary Fig. 1c, d). Next, ultrafine palladium nanoparticles of diameter around 1.3 nm were synthesized by in situ reduction and attached to the pores’ inner surfaces, as clearly shown in the SEM-EDX (Supplementary Fig. 2b), TEM, and elemental mapping images (Supplementary Fig. 3), powder X-ray diffraction (XRD) analysis (Supplementary Fig. 4) and diameter distribution analysis (Supplementary Fig. 5). For further modification, the SP was successfully functionalized with azobenzene group (ASP) and then combined with CD (CASP). The variation of C1s, N1s XPS (Supplementary Fig. 6), and the changes of Fourier transformation infrared spectroscopy (FTIR) spectrum (Supplementary Fig. 7) confirmed the conjugation of the azobenzene groups and CD. Besides, the amount of Pd loaded in DMSN was estimated by inductively coupled plasma mass spectrometry (Supplementary Table 1).

### Catalytic efficacy of SP nanoparticles in vial

**Fig. 2:**
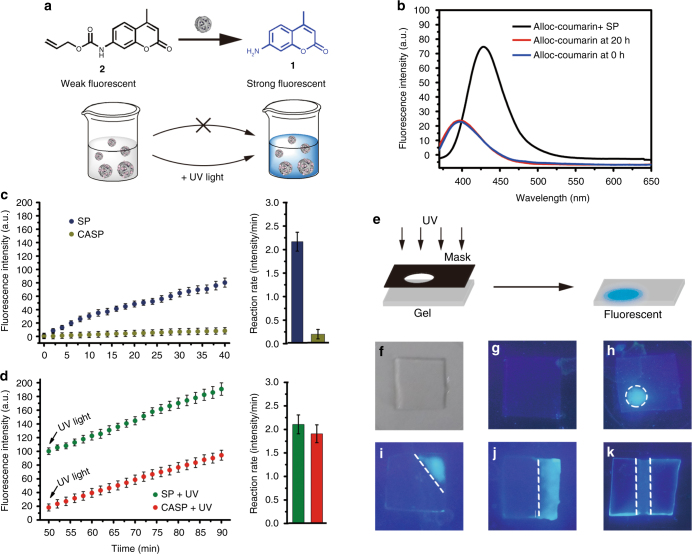
Catalytic efficacy of SP and CASP nanoparticles in vial. **a** SP and CASP catalyzed allylcarbamate cleavage of N-alloc-cumarin, **2** to generate a fluorescent compound, **1**, in vial. **b** The fluorescent spectra of alloc-coumarin alone at 0 h (blue) or 20 h (red) and reacted with SP for 20 h (black). **c** Fluorescence was increased when the system added SP, while CASP showed no significant change during 0–40 min. **d** After irradiation with UV light for 10 min, the catalytic activity of CASP was restored and became similar to SP, while UV light had no effects on SP. The attached graph reflected the reaction rates of SP and CASP before and after the UV light. Data were presented as mean ± s.d. (*n* = 3). **e** The fluorescent gel experiments: the polyacrylamide gel (PAMG) was prepared in the presence of CASP and N-alloc-coumarin (**f**). Under ambient conditions, the PAMG showed minimal fluorescence (**g**), when irradiated the gels with UV light under a mask that featured lower-left cycle (**h**), upper-right triangle (**i**), right rectangle (**j**), and middle rectangle (**k**)

As a prerequisite, we first validated the catalytic efficacy of SP in vitro by catalyzing the allylcarbamate cleavage of *N*-allyloxycarbonyl coumarin (N-alloc-cumarin), **2** (Fig. 2a). Comparing with the standard fluorescence spectrum of compounds **1** and **2** (Supplementary Fig. 8), the increased fluorescence intensity at 450 nm after incubation with SP (Fig. 2b) indicated the catalytic capability of SP. After 24 h, bright blue fluorescence was observed in the tube with SP. As expected, no reaction occurred in the tube with control DMSN particles (Supplementary Fig. 9). For further studying the catalytic efficiency and kinetic process between substrates and products, we recorded the changes of fluorescence spectrum from 0 to 9 h (Supplementary Fig. 10), and analyzed the supernatants of the reactions with high-performance liquid chromatography (HPLC) (Supplementary Fig. 11). The data showed clearly that the catalytic process was almost finished in about 9 h. The relating statistics of concentration of reagent and product and translation ratio were shown in Supplementary Fig. 12. The relationship between natural logarithm of reactant concentration and reaction time (ln[c]-t) indicated that the allylcarbamate cleavage of N-alloc-cumarin was belonged to the first-order reaction under our experimental condition. More importantly, the translation ratio of reagent was more than 95% at 9 h with our catalysts, while the valve <1% with reagent alone (Supplementary Fig. 12c). This indicated that the confinement effect of DMSN, excellent catalytic performance of Pd^0^ and large specific surface area of the ultra-small Pd nanopaticles synergistically improved the catalytic activity. Besides, the different reaction conditions had no obvious disturbance on the catalytic activity of SP (Supplementary Fig. 13 and Supplementary Table 2), showing the stability of this catalyst.

### Light-mediated reversible catalysis process

We next studied the light-gated bioorthogonal catalysis. For the light-gated process, the Azo modified on DMSN was one key factor in our design. The quantity of Azo modified on DMSN was estimated about 50 μg mg^−1^ (Supplementary Fig. 14). More importantly, the Azo modified on DMSN still retained its photo-responsive property (Supplementary Fig. 15). After treated with β-CD, the catalytic activity of CASP was inhibited because almost no fluorescent change was observed at 450 nm (Supplementary Fig. 16a). As expected, the catalyst could be activated by light: after irradiation by UV light, the catalytic activity was restored again (Supplementary Fig. 16a). Besides, the catalyst could be reset to its inactive state reversibly by visible light (Supplementary Fig. 16b). Kinetic studies further supported the light-activated catalysis (Fig. 2c, d). When the system was irradiated by UV light for 10 min (Fig. 2d), the fluorescence was increased suggesting the activation of CASP. Control studies of SP showed its catalytic activity was unaffected by light indicating the critical role of reversible interactions between Azo and CD. LC-MS observed the fluorescent reaction product in the solution with CASP treated by light (Supplementary Fig. 17). Therefore, we could control the chemical reaction to initiate or terminate by light at proper time through this system. Moreover, the spatial resolution of our light-mediated process was also confirmed with a fluorescent gel experiment (Fig. 2e)^39^. When re-activating the catalysts in thin polymer gels through irradiation locally under a mask, we might be able to create a corresponding fluorescent pattern. The polyacrylamide gel (PAMG) was prepared in the presence of CASP and compound, **2**, (Fig. 2f). Under ambient conditions, the PAMG showed minimal fluorescence (Fig. 2g). To demonstrate our supposition, we irradiated the gels with UV light under a mask that featured lower-left cycle (Fig. 2h), upper-right triangle (Fig. 2i), right rectangle (Fig. 2j), and middle rectangle (Fig. 2k). The fluorescence in the exposed regions turned on, which indicated fluorescent precursors were converted to fluorescent product. Besides, the heterogeneous nanocatalysts were recyclable with high catalytic activity (Supplementary Table 3). These results suggested that the light-gated transition metal catalysts had good spatial and temporal resolution.

### The light activation of CASP in living cells

Having confirmed the catalytic activity of nanocatalysts in solution, we next studied their intracellular performance in human cervical cancer cells (HeLa cells) as the model system (Fig. 1c). Before that, the cellular uptake of the nanocatalysts was quantified by tracking [^106^Pd] by ICP-MS, both SP and CASP could be effectively endocytosed by HeLa cells, respectively (Supplementary Fig. 18). In addition, according to the cytotoxicity experiments (Supplementary Fig. 19a), SP and CASP were biocompatible at the concentrations used in our study. Besides, the low-dose UV light did not cause cytotoxicity avoiding extra light damage (Supplementary Fig. 19b).

**Fig. 3:**
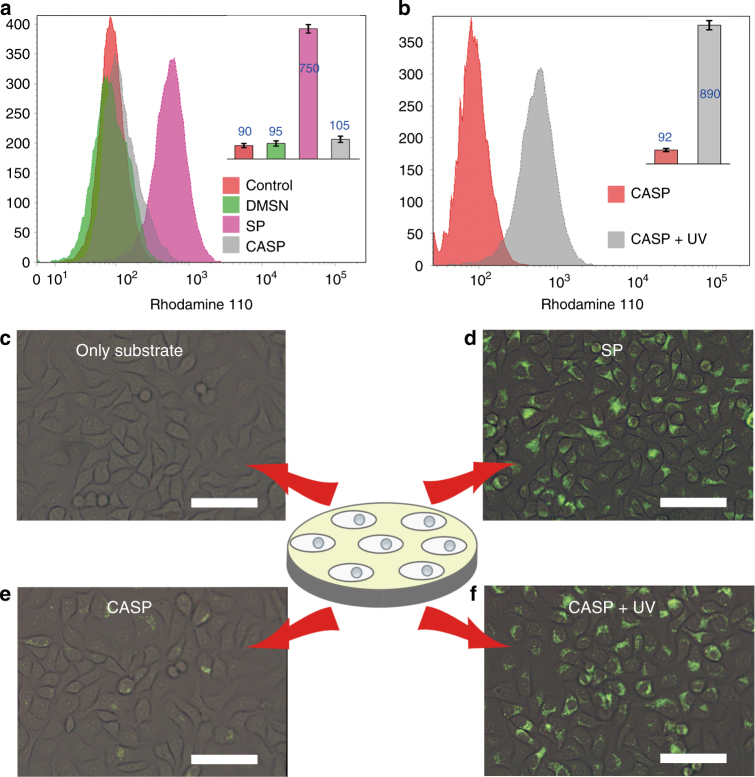
Triggered cleavage of alloc-Rhodamine 110 (**4**) in living cells. **a** The flow cytometry analysis of control (cells alone), DMSN, SP, and CASP. **b** Treated the CASP with UV light, the cells produced an increased fluorescence in the flow cytometry. **c**–**f** Fluorescence images of HeLa cells. A green fluorescence was observed with SP (**d**) and CASP + UV light (**f**), while no fluorescence was obtained with substrate only (**c**) and CASP (**e**) (scale bar = 50 µm). Data were presented as mean ± s.d. (*n* = 3)

Then, the catalytic activity of the complex catalysts was investigated in living cells. Transformation of rhodamine 110 derivative (**4**) into rhodamine 110 (**3**) was chosen as the model reaction^25^. The nanocatalysts were incubated with HeLa cells first, and then the old medium was replaced by the fresh one containing substrate, **4**. After 24 h, the cells were washed with phosphate buffer (PBS) for three times. The resulting fluorescent compound, **3**, was retained within the cell once the allylcarbamate cleavage took place (Fig. 1c). As shown in the flow cytometry analysis (Fig. 3a), a significant fluorescence increase was observed for the cells treated with SP in comparison with the controls. The results indicated that the nanocatalyst could still catalyze the reaction in cells. However, CASP showed little catalytic activity under this condition, indicating that CASP was stable in cells.

We next investigated the intracellular light-gated process of CASP. When treated the catalyst with UV light, CASP restored its catalytic activity with an obvious fluorescence increase (Fig. 3b). The increased catalytic efficiency was echoed in fluorescence imaging (Fig. 3c–f). We synchronously monitored the intracellular catalytic reactions at first 1 h, the relatively steady reaction rate suggested the stable catalytic activity of CASP in living cells after treated by UV light (Supplementary Fig. 20) and the recycle experiment indicated that in short term our system could realize the reversible activation of TMCs in cells more than five cycles (Supplementary Fig. 21). The fluorescence images showed similar results (Supplementary Fig. 22). What’s more, after the activation of the catalyst by UV light, if we treated the system with visible light again, the catalyst showed no activity once more (Supplementary Fig. 22c). Certainly, the UV and Vis light had no obvious effect on the catalytic activity of SP showing the activity switch was due to the interaction between Azo and CD (Supplementary Fig. 23). Also, the LC-MS analysis of the final product (331.10) of the bioorthogonal reaction catalyzed by CASP ensured that the catalytic reaction was carried out correctly (Supplementary Fig. 24).

### Light-gated synthesis of a mitochondria-specific probe

**Fig. 4:**
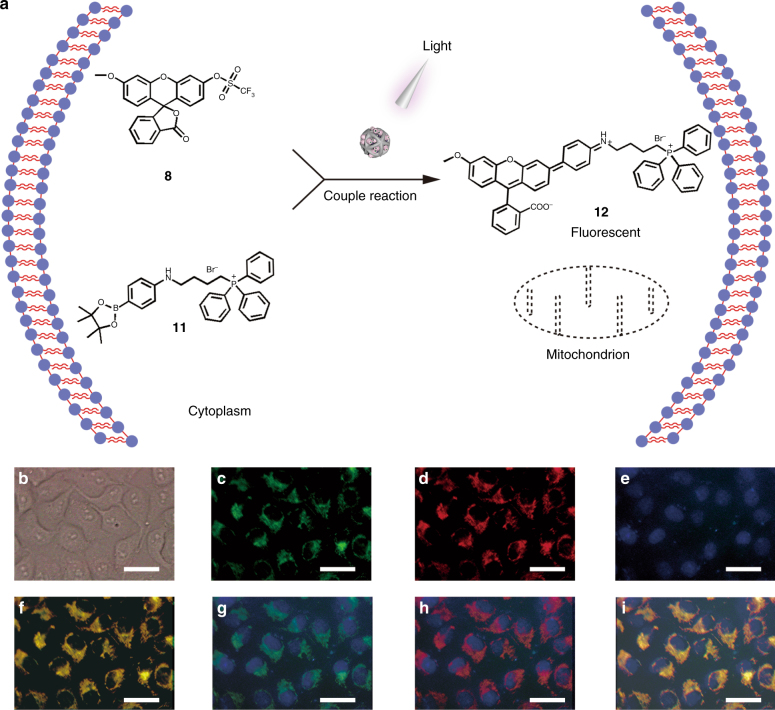
Light-gated Suzuki–Miyaura coupling reaction. **a** Scheme of the light activation^10^ of CASP catalyzed cross-coupling reaction generates the mitochondria-localized fluorescent product **12**. **b-i** Collection of the fluorescence images of the CASP catalyzing the Suzuki–Miyaura coupling reaction with the UV light activation: **b** bright-field image of HeLa cells, **c** emission of the compound **12** (green channel), **d** the mitochondrion labeled with MitoTracker^®^ Red CMXRos (red channel), **e** the cell nucleus labeled with Hoechst 33258 (blue channel), **f** merging with **c** and **d**, **g** merging with **c** and **e**, **h** merging with **d** and **e**, **i** merging with **c**, **d**, and **e** (scale bar = 10 µm). The low fluorescence intensity of green channel indicated the low catalytic activity of CASP as we designed

In addition to the cleavage reaction, we explored the possibility of catalyzing other types of reactions. Among them, cross-coupling reactions play an important role in chemical synthesis of molecules^40^. The cross-coupling reaction under mild conditions especially attracts attention^41^. To explore the formation of the C–C cross-coupled product in cells, we decide to synthesize a fluorescent dye based on the palladium-mediated synthesis via Suzuki–Miyaura cross-coupling reaction (Fig. 4a)^10^. The non-fluorescent compounds, **8** and **11**, can produce fluorescent target product **12** through the couple of triflate and phenylboronate groups. Meanwhile, since the product **12** has a triphenylphosphonium moiety, it can act as a mitochondria-specific fluorescent probe^11,42^. The light-gated synthesis of Suzuki–Miyaura probe **12** was initiated by incubation of the nanocatalysts with HeLa cells. After washing with PBS, the two reagents **8** and **11** were added. Then, we treated the cells with UV light. After another 24 h, the cells were labeled with a common mitochondrial red dye (MitoTracker Red CMXRos Ex/Em ≈ 579/599 nm) and Hoechst 33258 (nuclei stain) as controls. Cellular fluorescence was investigated by fluorescence images and flow cytometry. As shown in Fig. 4b–i, upon activation by UV light, the fluorescence images of synthesized dye (Fig. 4c) showed an obviously increased fluorescence co-localized with MitoTracker (Fig. 4d) when compared with the controls (Supplementary Fig. 25). The flow cytometry showed similar results indicating the light-gated catalytic reaction occurred (Supplementary Fig. 26). Besides, the two-channel flow cytometry analysis verified the independence between probe **12** and MitoTracker, eliminating mutual interference (Supplementary Fig. 27). These results demonstrated that the Suzuki–Miyaura coupling reaction could be also activated by light as expected. The fluorescent spectra of the reaction in solution and LC-MS of the products provided further supports (Supplementary Figs. 28–30). All results proved that light-gated Suzuki–Miyaura reaction could be realized successfully in living cells.

### Intercellular light activation of prodrug by the catalyst

Furthermore, we also carried out the intracellular conversion of prodrug 5-fluoro-1-propargyl-uracil (Pro-5FU) into 5-fluorouracil (5FU) by using our system (Supplementary Fig. 31). As a preliminary experiment, the toxicity profiles of Pro-5FU and 5FU were studied at various concentrations (Supplementary Fig. 31b). Compared with Pro-5FU, 5FU showed obvious toxicity with increasing the concentration. For the experiment of light-gated activation of Pro-5FU, the relative cell viability studies were carried out in the presence of the catalysts. As shown in Supplementary Fig. 31c, the cells that were incubated with CASP + UV light showed increased toxicity at high concentration of Pro-5FU, while CASP alone retained high cell viability. These results indicated that the toxicity was due to light-gated conversion of Pro-5FU into 5FU, but not the catalyst itself. Therefore, our system could be used in therapeutic applications such as the activation of multiple prodrugs on site of action.

## Discussion

We have fabricated a light-gated heterogeneous transition metal catalyst, which is capable to artificially control the bioorthogonal reactions in living cells. The ultrafine Pd nanoparticles deposited in DMSN motif have excellent catalytic activity in Pd-mediated bioorthogonal reactions. The light-gated host–guest interactions between azobenzene headgroup and β-CD play a critical and important role in reversibly regulation of the catalytic activity. This work not only provides new perspective to customize different heterogeneous catalysts with novel functions, but also has a huge potential in activation of prodrugs and synthesis of active molecules for precision therapy. Selective activation of bioorthogonal catalysts on demand can minimize the interference of organisms and realize multiple chemical reactions in situ. Our design is versatile and adaptable to a variety of chemical reactions in living cells for precision imaging and therapy.

## Methods

### Reduction of palladium in DMSN motif

For synthesis of SP nanoparticles, [PdCl_4_]^2^^−^ ions were absorbed onto the inner pore surfaces by coordinating and electrostatic interaction with amino groups. After in suit reduction, [PdCl_4_]^2^^−^ ions were converted to Pd^0^ leading to growing of palladium nanoparticles. The resulting nanoparticles DMSN (0.1 g) were dispersed in distilled water (10 mL) along with sonication for 30 min, followed by addition of H_2_PdCl_4_ (0.1 mL, 1 M) solution in 2 mL distilled water, after 20 min, a freshly prepared NaBH_4_ (36 mg in 4 mL cold water) was added into the above aqueous solution under vigorous stirring. After that, the resulting suspension was stirred for another 3 h. Finally, the mixture was centrifuged at 11,000 × *g* for 10 min to separate the SP. Then, SP was washed by water three times and dried under vacuum.

### The fluorescent gel experiments

The polyacrylamide gel (PAMG) was prepared via radical-copolymerization of acrylamine in the presence of CASP and N-alloc-coumarin. Aliquots of 750 mg acrylamide (AAm) and 25 mg Bis-acrylamide (MBA) were dissolved in 2.5 mL distilled water as solution **1**. The CASP (100 µg mL^−1^) and 10 µM N-alloc-cumarin (40 mM in dimethyl sulfoxide (DMSO)) were mixed in 2.45 mL distilled water as solution **2**. We mixed solution **1** and solution **2** to obtain a uniformly dispersed solution and added 40 µL 10% ammonium persulphate (APS) and 10 µL tetramethylethylenediamine (TEMED) to the mixture. Then, the mixture was transferred to the mold and reacted for 2 h at room temperature. The prepared PAMG was cut into square (1 cm × 1 cm) and irradiated with UV light for 10 min under the different masks that featured lower-left cycle, upper-right triangle, right rectangle, and middle rectangle. After 20 h, the fluorescent images were recorded by a camera on the UV transilluminators.

### Light-gated catalytic reactions in living cells

HeLa cells were cultured with Dulbecco’s modified Eagle medium (DMEM) containing fetal bovine serum (10%) at 37 °C under a humidified atmosphere with 5% CO_2_. For flow cytometry analysis, HeLa cells were seeded at around 60,000 cells per well in 6-wells plates for 24 h prior the experiment. Thereafter, the nanocatalysts SP or CASP (40 µg mL^−1^) were incubated with pre-seeded HeLa cells, respectively. After 24 h incubation, old media was removed and cells were washed three times with PBS to eliminate extracellular nanoparticles. Then, the cells were treated with 0.12 W cm^−2^ UV light for 10 min. Protected rhodamine 110, **4** (10 mM in DMSO) was added to the culture at final concentration of 20 µM and incubated for 24 h. After that, cells were rinsed with PBS twice, harvested with trypsin, and resuspended in PBS buffer. The intracellular presence of fluorescent compound, **3**, was analyzed by flow cytometry using FITC-like band pass emission filters (530/30).

### Suzuki–Miyaura coupling reaction in living cells

The cells were incubated on sterilized cover slips in a 24-wells plate at 37 °C. Old media was removed and replaced by fresh media containing SP or CASP (40 µg mL^−1^) and incubated for another 24 h. Excess of extracellular nanoparticles was removed by washing with PBS buffer thrice. Compounds **8** and **11** (20 mM in DMSO) were diluted in fresh media to give a final concentration of 20 µM and incubated at 37 °C for 24 h. After that, the cells were washed with PBS twice, subsequently, mitochondria were stained by 20 min incubation with 50 nM of MitoTracker^®^ Red CMXRos at 37 °C. The nuclei were stained by incubation with a 10 µg mL^−1^ solution of Hoechst 33258 in media for 15 min. The fluorescence images were collected.

### Data availability

All data are available from the authors on reasonable request.

## Electronic supplementary material

Supplementary Information(PDF 3974 kb)
