# Supplementary material for: Designed heterogeneous palladium catalysts for reversible light-controlled bioorthogonal catalysis in living cells
Source: Nat Commun. 2018 Mar 23;9:1209. doi: 10.1038/s41467-018-03617-x (PMC5865172; doi:10.1038/s41467-018-03617-x)
Supplement: Supplementary file 1 — Supplementary Information(PDF 3974 kb) [file 41467_2018_3617_MOESM1_ESM.pdf]

Designed Heterogeneous Palladium Catalysts for Reversible Light-controlled Bioorthogonal Catalysis in Living Cells

Wang et al.

List of Contents

***Supplementary Methods.**

***Supplementary Note 1. Synthesis and Characterization of CASP**

***Supplementary Note 2. CASP Mediated Allylcarbamate Cleavage in Vial**

***Supplementary Note 3. Light Activation of CASP inside Living Cells**

***Supplementary Note 4. Light-Mediated Suzuki-Miyaura Coupling Reaction to Synthesize A Mitochondrial Target Probe**

Supplementary Note 5. Light-activated Pro-5FU to synthesize anticancer drug *in situ

***Supplementary Note 6. Synthesis of Chemical Substrate Molecules in This Study**

***Supplementary References**

Supplementary Methods.

Reagents and materials

Tetraethylorthosilicate (TEOS), cetyltrimethylammonium tosylate (CTATos) and sodium hydroxide (NaOH) were purchased from Sigma-Aldrich. Hydrogen tetrachloropalladate (Ⅱ) (H_2PdCl_4) were obtained from Sinopharm Chemica1 Reagent Co. (Shanghai, China). Sodium borohydride ($NaBH_4$), β-Cyclodextrin (CD), 4-aminoazobenzene (Azo-NH_2), methyl iodide, triethanolamine, and succinic anhydride were from Aladdin. acrylamide (AAm), Bis-acrylamide (MBA), Ammonium persulphate (APS) tetramethylethylenediamine (TEMED), fluorescein, *N*-phenyl-bis(trifluoromethanesulfonimide), Fluorescein isothiocyanate (FITC), (4-bromo butyl)triphenylphosphonium bromide , 4-Aminophenylboronic acid pinacol ester, Rhodamine 110, allylchloroformate, 7-Amino-4-methylcoumarin and 3-aminopropyltriethoxysilane (APTES) were obtained from Alfa Aesar. NaH_2PO_4, Na_2HP_4, HCl and H_2O_2 were obtained from Beijing Chemicals (Beijing, China). MitoTracker Red CMXRos was from Sangon Biotechnology Inc. (Shanghai, P.R. China). All other reagents were of analytical reagent grade, and used as received. Ultrapure water (18.2 MΩ; Millipore Co. USA) was used throughout the experiment.

Apparatus and characterization

UV-Vis absorbance measurement was carried out on a JASCO V-550 UV-Vis spectrophotometer. Fluorescence spectra were detected by JASCO F-6000 fluorescence spectrometer with a Peltier temperature control accessory. FT-IR spectra were carried out on a BRUKE Vertex 70 FT-IR spectrometer. Scanning electron microscopic (SEM) images were recorded using a Hitachi S-4800 Instrument (Japan). Transmission electron microscopic (TEM) images of cells were captured with a FEI TECNAI G2 20 high-resolution transmission electron microscope operating at 200 kV. N_2 adsorption-desorption isotherms were recorded on a Micromeritics ASAP 2020M automated sorption analyzer. The pore size was determined following the BJH method. The TGA-DSC analysis was recorded by STA 449 F3 Jupiter simultaneous thermal analyzer. ^{1}H NMR spectrum was recorded on a Bruker-600 MHz NMR instrument. The crystalline structures of the as prepared samples were evaluated by X-

ray diffraction (XRD) analysis on a Rigaku-Dmax 2500 diffractometer by using CuKα radiation. X-ray photoelectron Spectroscopy (XPS) spectra were analyzed by Thermo Fisher Scientific ESCALAB 250Xi Spectrometer Electron Spectroscopy (America). ICP-MS measurements were performed on a TheroScientific Xseries Ⅱ inductively coupled plasma mass spectrometer. The flow cytometry data were obtained by BD LSRFortessa™ Cell Analyzer. Microwave reactions were carried out by Biotage Initiator+ Microwave System.

Supplementary Note 1.

Synthesis of dendrimer-like macroporous silica nanoparticles (DMSN)

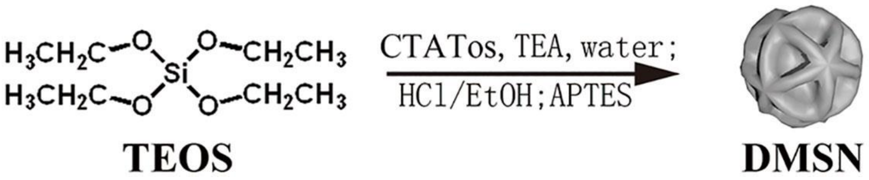

The DMSN were prepared according to the literature with little modification.[1] In brief, a mixture of cetyltrimethylammonium tosylate (CTATos, 1.92 g), triethanolamine (0.31 g) and water (100 mL) was stirred at 80 °C for 1 h, then 15.6 mL TEOS was added and the mixture was stirred at 80 °C for another 2 h. The synthesized DMSN were filtered, washed with water and ethanol and dried in oven at 60 °C for 12 h.

To modify the DMSN inner and outer surfaces with amino groups, the surfactant template CTATos in the pores of DMSN were removed first. The as-synthesized DMSN were dispersed in a solution of hydrochloric acid in ethanol (10 % V/V) and extracted at 78°C for 24 h. This process was repeated for three times. Afterwards, 500 mg of the template-remove DMSN were dispersed in 50 mL toluene by sonication and 200 µL (3-aminopropyl) triethoxysilane were added to the suspension prior to refluxing at 113°C under N_2 for 12 h. Thus, DMSN were aminated and then collected after centrifugation, washing with ethanol and drying under vacuum.

Deposition of Pd nanoparticles in the pores of the DMSN (SP)

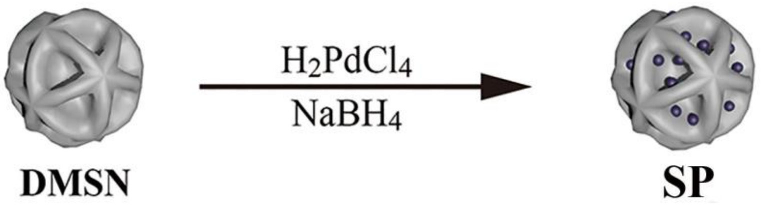

For synthesis of SP, $[PdCl_4]^{2-}$ ions were absorbed onto the inner pore surfaces by coordinating and electrostatic interaction with amino groups. After in-suit reduction by $NaBH_4$, $[PdCl_4]^{2-}$ ions converted to Pd^0 leading to aggregation and the generation of Pd^0 nanoparticles.[2] Ultrafine and well-dispersed Pd^0 nanoparticles with diameter of 1-2 nm were formed and simultaneously attached to the inner surface. Typically, the aminated DMSN nanoparticles (100 mg) were dispersed in 10 mL distilled water by sonication for 30 min, followed by the addition of the H_2PdCl_4 (0.1 mL, 1M) diluted in 2 mL distilled water. After 20 min, a freshly prepared $NaBH_4$ (36 mg in 4 mL cold water) was added into the above aqueous solution under vigorous stirring. After mixture, the resulting suspension was stirred for another 3 h. Finally, the suspension was centrifuged at 10000 rpm for 10 min to separate the SP. Then, SP was washed by water 3 times and dried under vacuum.

Preparation of azobenzene terminated SP (ASP)

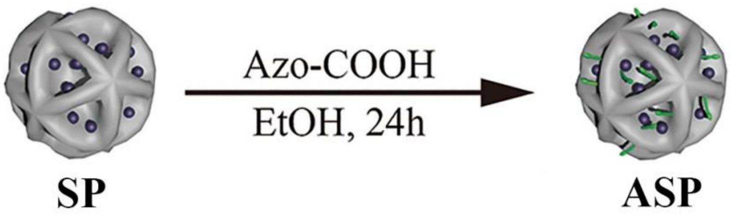

The synthesis of ASP was according to the literature with a little modification.[3] The 40 mg as-prepared SP was dissolved in 8 mL anhydrous ethanol and the solution was sonicated to disperse nanoparticles. The 8 mg Azo-COOH was added into the mixture and sonicated 5 min. The solution was then allowed to stir under room temperature for >24 h under dark. The nanoparticles were separated from solution by centrifugation and washed with water and ethanol respectively. The particles were dried under vacuum. The conjugation of the azobenzene groups on surface of DMSN was confirmed by X-ray photoelectron spectroscopy (XPS) and Fourier transformation infrared spectroscopy (FTIR) spectrum.

Capping ASP with β-cyclodextrin (CASP)

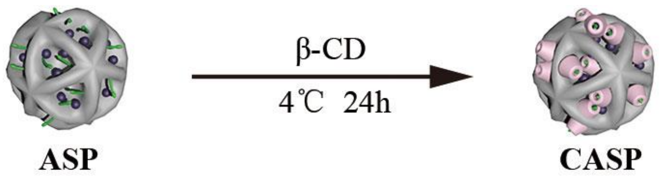

The dried 20 mg ASP was dissoved in 10 mL distilled water firstly, then β-CD was added into the solution. The mixture was then stirred and sonicated. After complete dessolvation of CD, the solution was allowed to stir at 4 °C > 24 h to maximize the association of CD and the formation of the pseudorotaxanes. After that, the nanoparticles were separated from solution via centrifugation and then washed using 2 mM CD solution. The product was separated from washing solution by centrifugation. The nanoparticles were subsequently dried via vacuum overnight for analysis.[3]

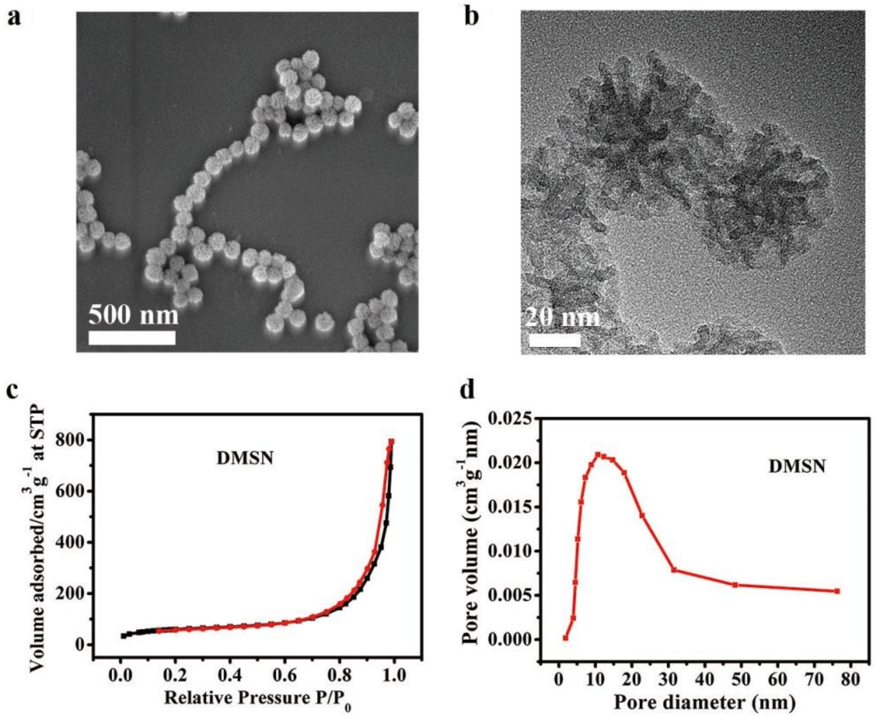

Supplementary Figure 1. (a) The SEM image of the obtained DMSN (scale bar = 500 nm). (b) The TEM image of the obtained DMSN (scale bar = 20 nm). The N_2 adsorption–desorption isotherms (c) and the corresponding pore-size distribution curve (d) indicate the mean pore size of DMSN is 12.3 nm.

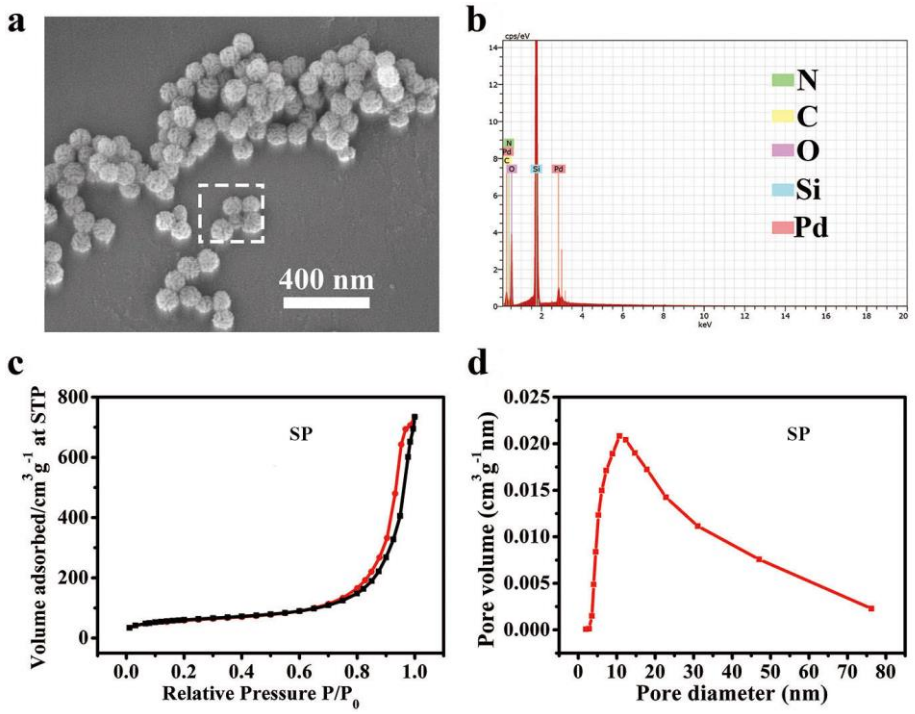

Supplementary Figure 2. (a) The SEM image of the obtained SP (scale bar = 400 nm). (b) The SEM-EDX analysis of the obtained SP (around the dashed box), indicated that the Pd nanoparticles were formed in the DMSN successfully. The N_2 adsorption–desorption isotherms (c) and the corresponding pore-size distribution curve (d) indicated the Pd reduction didn't destroy the structure of DMSN.

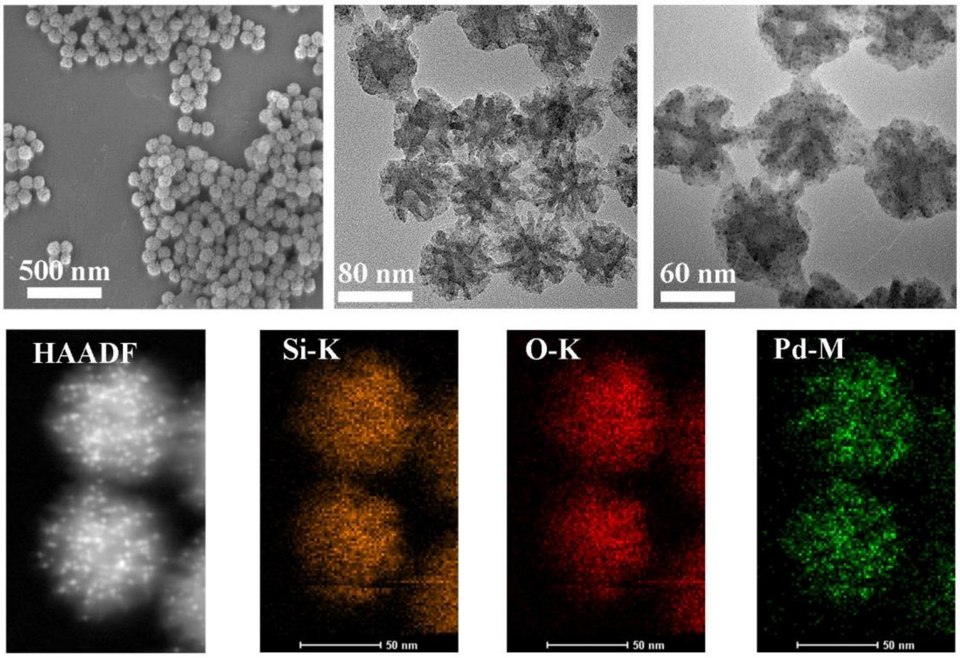

Supplementary Figure 3. The TEM images of the obtained SP. The element mapping of Si-K, O-K and Pd-M indicated the palladium nanoparticles evenly distributed in DMSN.

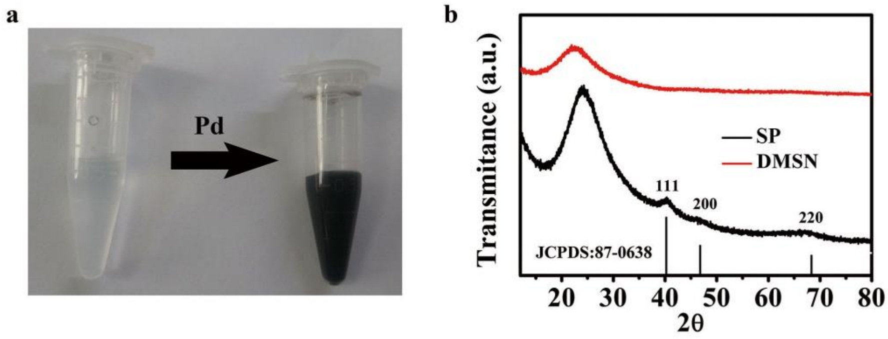

Supplementary Figure 4. (a) The photograph of the DMSN before (left) and after (right) reducing Pd in situ. An obvious brownish black color was appeared in the product of SP. (b) The XRD pattern also proved the formation of PdNPs on silica and the structure of DMSN was remained (Pd: JCPDS Card NO. 87-0638).

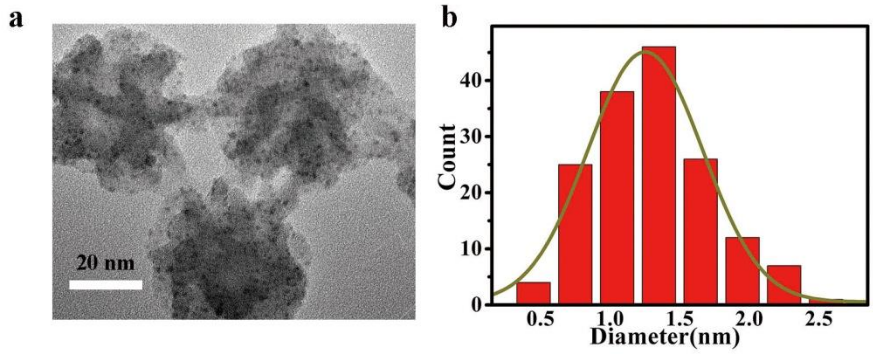

Supplementary Figure 5. (a) High-resolution TEM images of SP (scale bar = 20 nm). (b) Size distribution histogram of SP. The total number of clusters counted for the histogram was 200. The average diameter of the PdNPs on the silica was 1.3 nm.

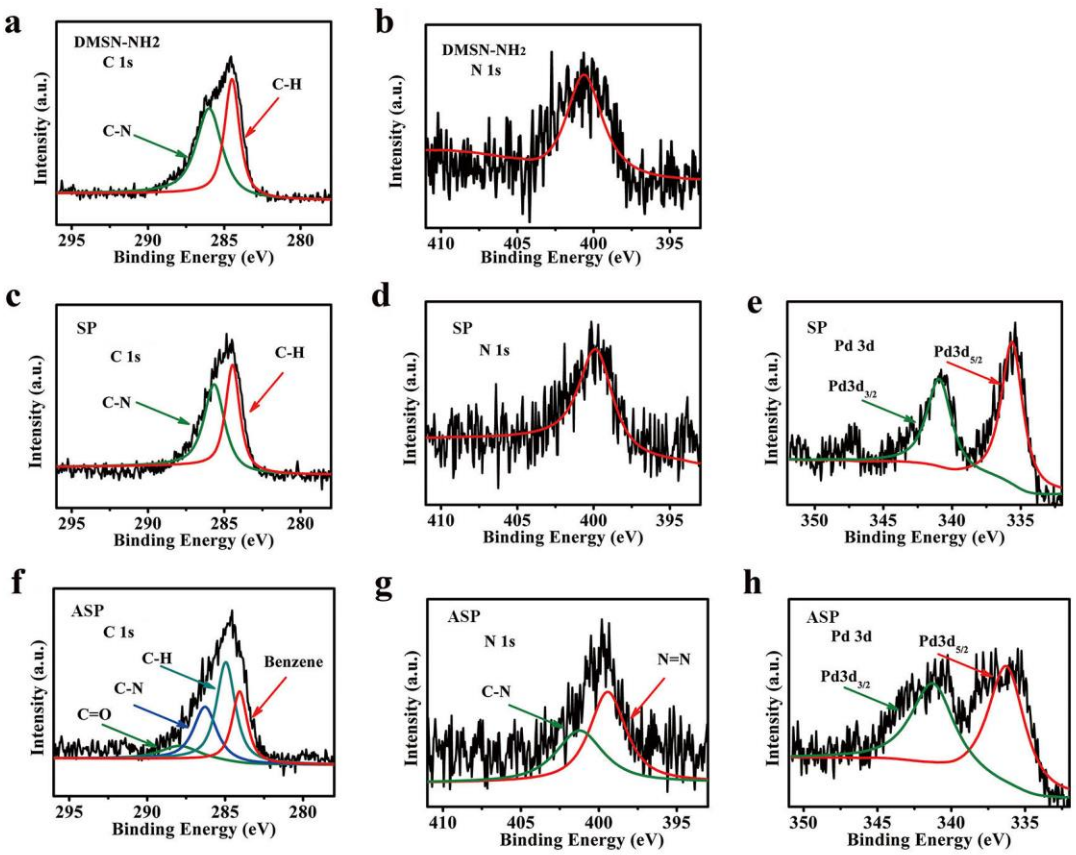

Supplementary Figure 6. X-Ray Photoelectron Spectroscopy (XPS) spectra of DMSN, SP and ASP. (a) C1s core level and (b) N1s core level spectra of the obtained aminated DMSN. (c) C 1s core level, (d) N 1s core level and (e) Pd 3d core level spectra of the as-synthesized SP. (f) C 1s core level, (g) N 1s core level, and (h) Pd 3d core level of ASP. The Pd 3d XPS spectrum indicated that the Pd nanoparticles actually existed in obtained SP. In comparison with the C 1s XPS spectrum of ASP, the appearance of the C=O and benzene group confirmed the successful modification of azobenzene.

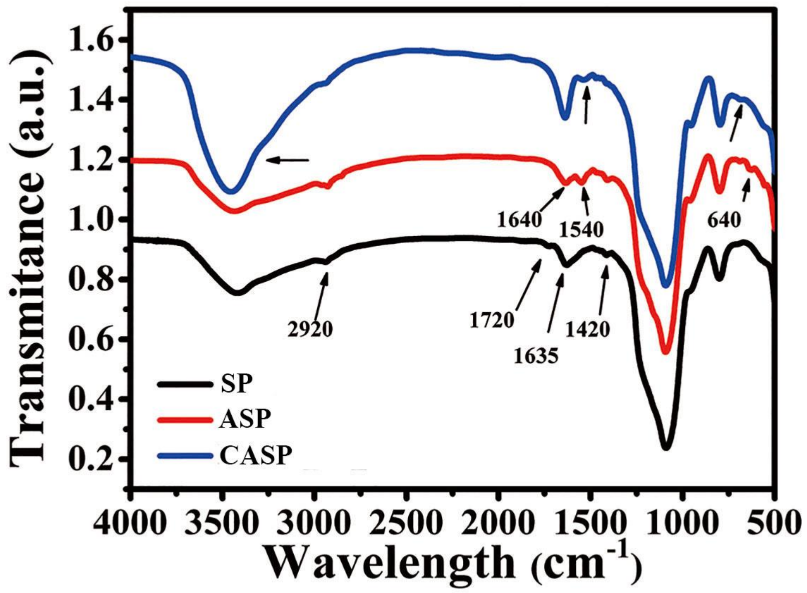

Supplementary Figure 7. The FTIR spectra of the SP (black), ASP (red) and CASP (blue). As for SP (black), a weak band at 2936 cm^{-1} was ascribed to symmetric vibration of the C-H groups, and a band at 1550 cm^{-1} was the bending vibration N-H, indicating the presence of NH_2 in SP; for ASP (red), the 1640 cm^{-1} was the C(=O)NHR stretching, 1540 cm^{-1} was NH stretching and the bene skeletal vibration, and 640 cm^{-1} was the ring deformation of bene, indicated the Azo has been successfully introduced on the surface of ASP; for CASP (blue), the decrease of band 1540 cm^{-1} and 640 cm^{-1} was owing to the interaction between CD and Azo, the band at 3500 cm^{-1} became wider was originated from the intramolecular association of O-H in cyclodextrins, this results comfirmed that the CD could combine with Azo sucessfully under our experiment conditions.

Inductively coupled plasma mass spectrometry (ICP-MS) instrumentation for quantification of SP

ICP-MS measurements were performed on a TheroScientific Xseries Ⅱinductively coupled plasma mass spectrometer. 0.5 mL of fresh aqua regia was added to the 10 μL sample solution and then the sample was diluted to 10 mL with distilled water. The amount of ^{106}Pd and ^{28}Si were tracked by ICP-MS. The ^{28}Si was come from SiO_2 and ^{106}Pd was from Palladium nanoparticles. Therefore, The Pd load amount in DMSN was estimated with the following formula:

$$Pd\ load\ \% = M_1/[M_1+M_2/28.02*(28.02+32)]*100\%$$

M_1: The amount of ^{106}Pd tracked by ICP

M_1: The amount of ^{28}Si tracked by ICP

Supplementary Table 1 Pd amount in SP calculated by ICP-MS

Pd (ppm)	Si (ppm)	Pd/Si	Pd load %
5.608	28.200	0.199	8.49
6.180	33.300	0.186	8.01
5.904	30.200	0.195	8.37

Supplementary Note 2.

SP induced allylcarbamate cleavage inside vial

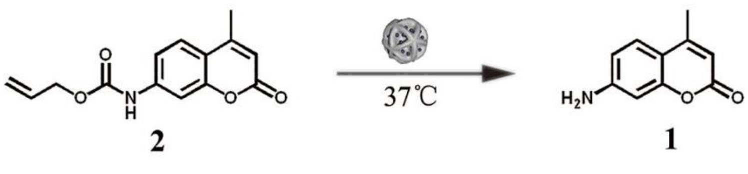

The allylcarbamate cleavage of N-alloc-coumarin, **2**, in vitro was carried out to assess the catalytic efficiency of SP and CASP. Brifely, the SP (100 μg mL^{-1}) and 10 μM N-alloc-coumarin (40 mM in DMSO) were mixed in water, PBS and DMEM medium respectively at 37 °C. After a period of time, the mixture was centritigated to recover

the nanocatalysts. The changes of fluorescence of the supernatant were detedcted by fluorescence spectrometer. The fluorescence performance of **2** and **1** was also shown in Figure S8.

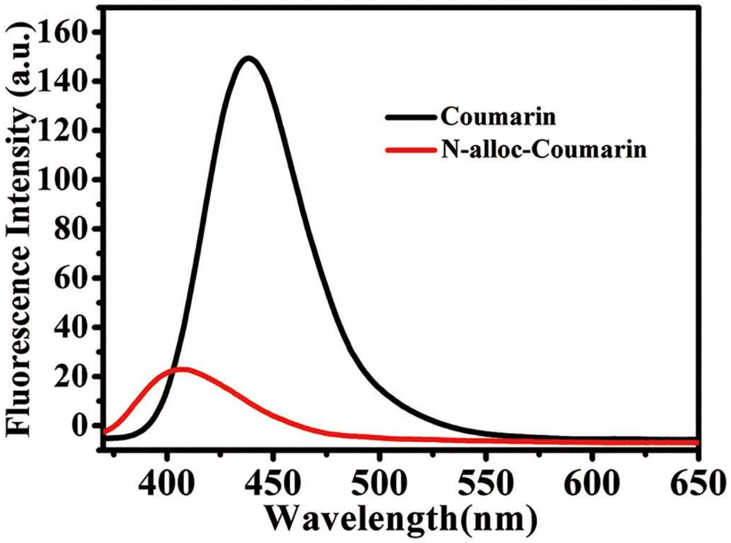

Supplementary Figure 8. The standard fluorescence spectrum of N-alloc-coumarin, **2** (red) and coumarin, **1** (black). The changes of fluorescence indicated that the SP could catalyze allylcarbamate cleavage reaction.

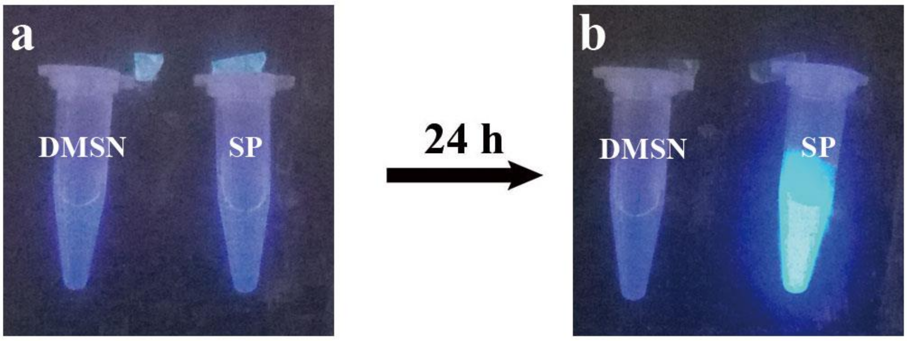

Supplementary Figure 9. Photos of the reaction mixtures in water with DMSN and SP under room temperature at (a) 0 h and (b) 24 h.

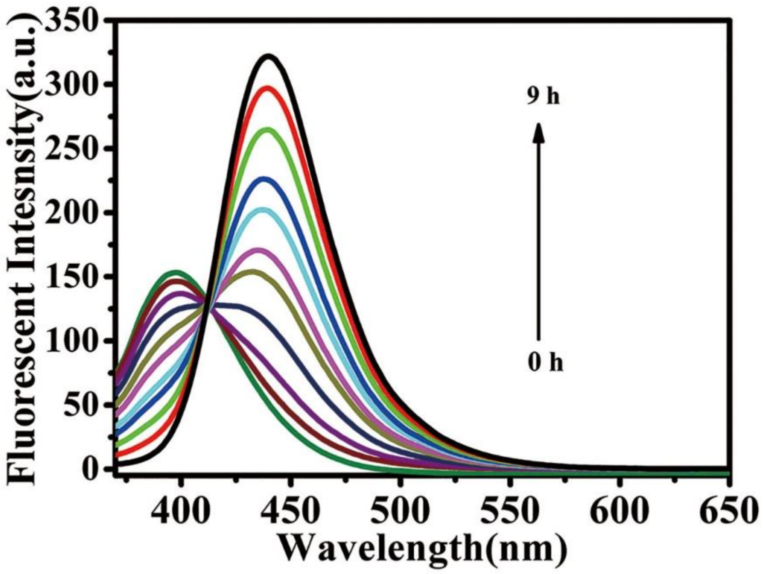

Supplementary Figure 10. The fluorescence spectra of N-alloc-coumarin allowing at different reaction time (0 - 9 h).

HPLC study of allylcarbamate cleavage in solution

Analytical HPLC with absorption detection at λ=280 nm. Eluent A: 0.05 M ammonium acetate containing acetate acid (0.1 %); Eluent B: acetonitrile, formic acid (0.1 %) r_t = 3.5 min (A/B = 70 : 30 to 5 : 95 in 13 min, isocratic 1 min, 5 : 95 to 70 : 30 in 5 min, isocratic 1 min).

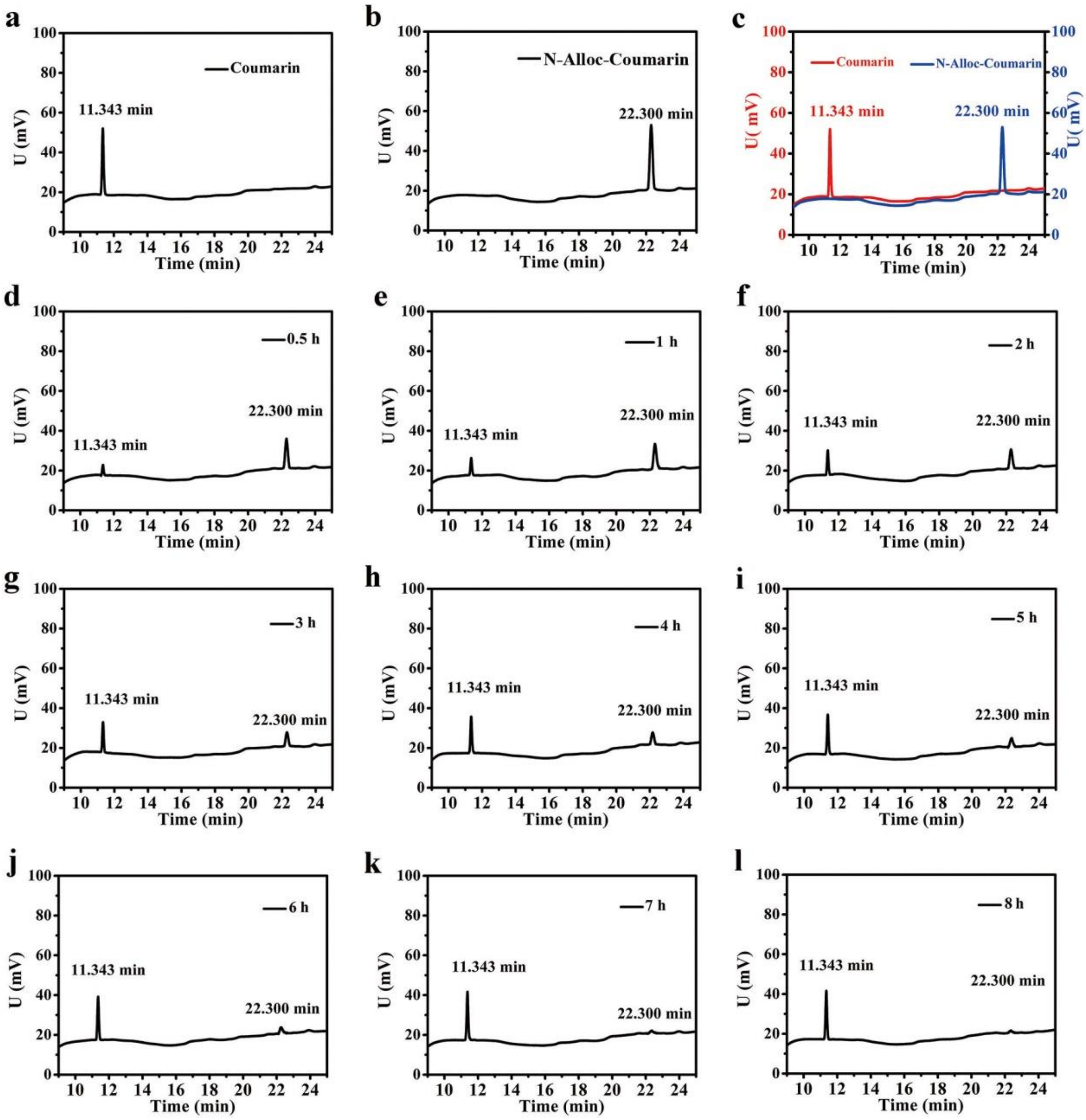

Supplementary Figure 11. The HPLC analysis of reaction process catalyzed by SP at different times. (a) - (c) The standard examples of coumarin and N-alloc-coumarin. (b) - (l) The examples of catalytic reaction at 0.5 - 8 h.

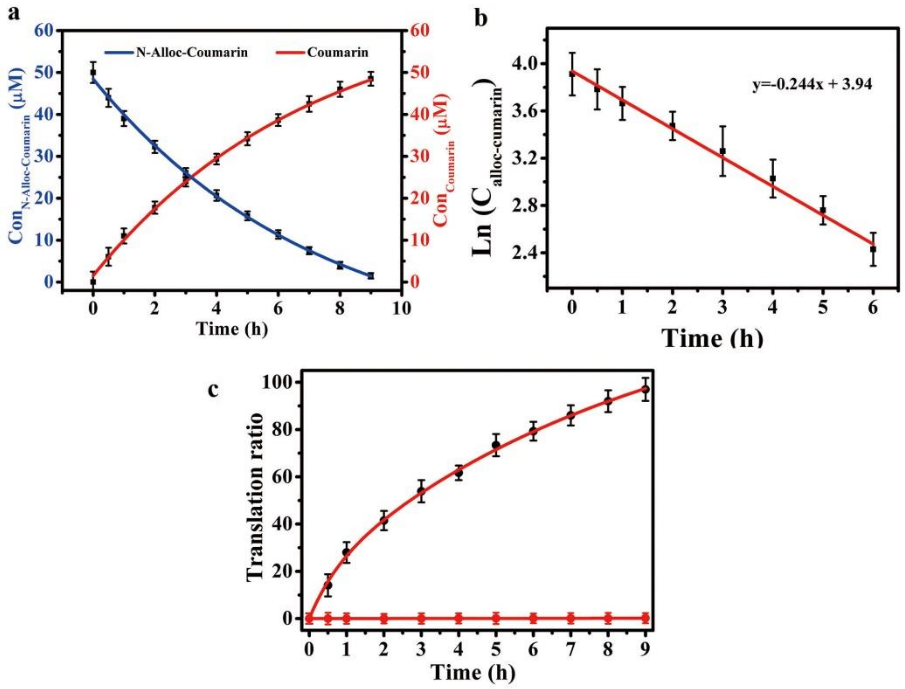

Supplementary Figure 12. (a) The decrease of N-alloc-coumarin and the increase of coumarin along with time with SP were shown according the HPLC analysis. (b) The diagram of the relationship between natural logarithm of reactant concentration and reaction time. (c) The translation ratio statistics of N-alloc-cumarin with (balck) or without (red) catalysts. Data were presented as mean ± s.d (n=3).

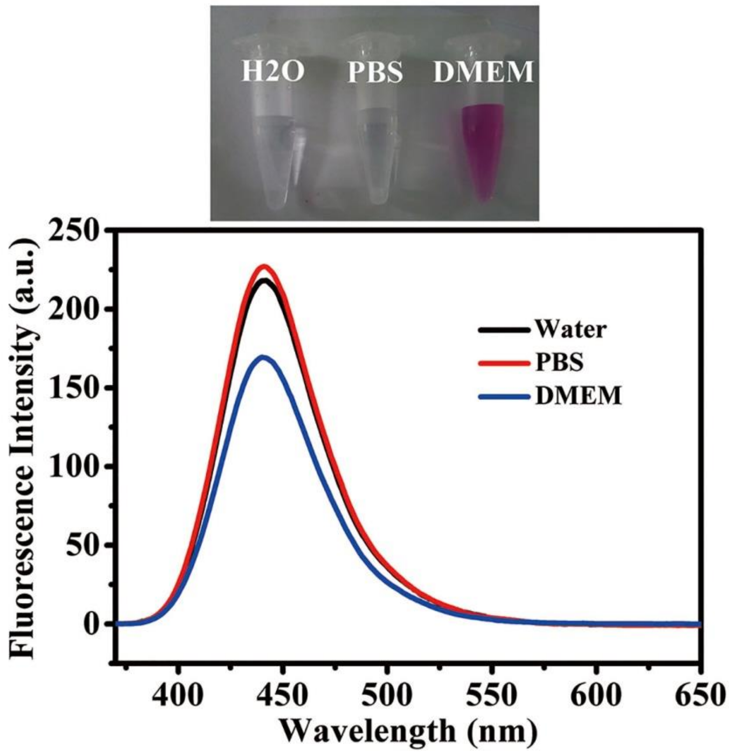

Supplementary Figure 13. The fluorescence spectra of the same concentration coumarin in water, PBS and DMEM. The fluorescence intesnsity of coumarin in PBS was close to that in water. Though, the fluorescence intensity of coumarin in DMEM decreased a little, the fluorescence performance of coumarin didn’t be disturbed by DMEM, which still could be used to monitor the reaction process.

Supplementary Table 2 Allylcarbamate cleavage experiments in different conditions

entry	solvent	N-alloc-coumarin	SP	CASP	Yield of 2
1	water	-	-	-	n/a
2	PBS	-	-	-	n/a
3	DMEM	-	-	-	n/a
4	water	+	-	-	0
5	PBS	+	-	-	0
6	DMEM	+	-	-	0
7	water	+	+	-	98
8	PBS	+	+	-	96
9	DMEM	+	+	-	96
10	water	+	-	+	6
11	PBS	+	-	+	5
12	DMEM	+	-	+	5
13	water	+	-	+*	91
14	PBS	+	-	+*	90
15	DMEM	+	-	+*	90

In the groups labeled with *, the CASP was treated with UV light.

Light-gated catalysis of SP and CASP in solution

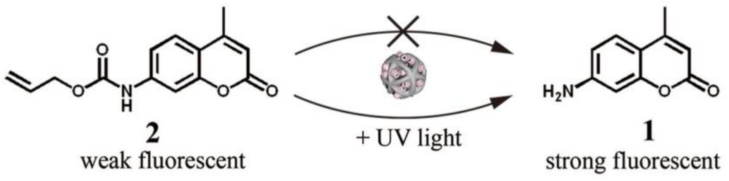

The catalysts SP or CASP (100 μg mL^{-1}) were mixed with 10 μM of N-alloc-coumarin (40 mM in DMSO) in water at 37 °C. Then, the mixtures were irradiated with UV light or Vis light for 10 min. After 12 h, the solutions were centritigated to recover the nanocatalysts. The changes of fluorescence of the supernatants were detedcted by fluorescence spectrometer. The fluorescence changes indicated that the

UV light had no effect on the SP, but could gate the catalytic process of CASP (Fig. S16a). Besides, the Vis light played an oppsite role to UV light (Fig. S16b).

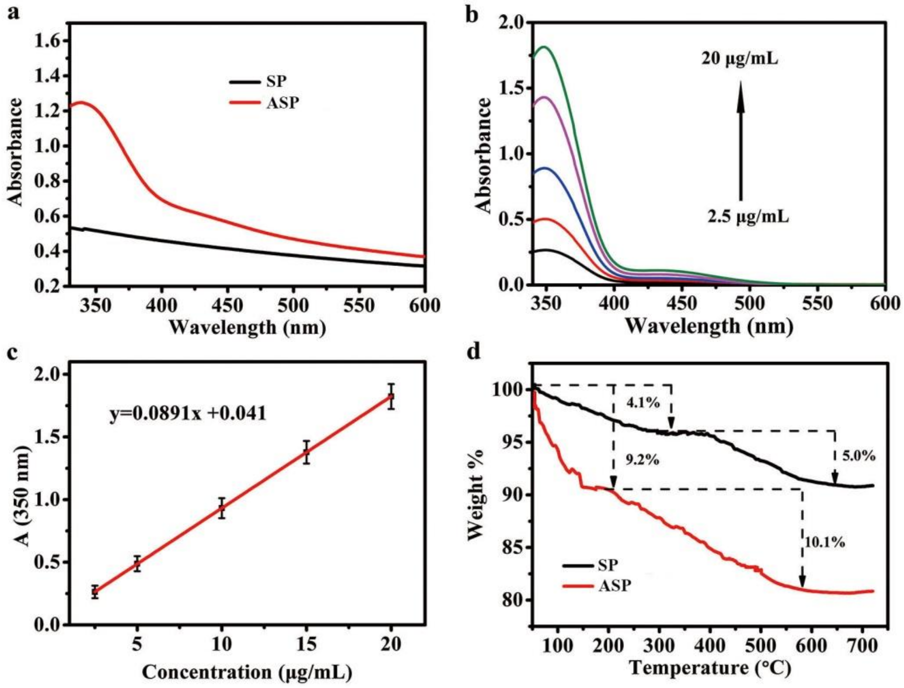

Supplementary Figure 14. (a) The UV-Vis spectra of SP and ASP (250 μg mL^{-1} methanol solution). (b) The UV-Vis spectrum of Azo-COOH (2.5, 5, 10, 15, 20 μg mL^{-1}). (c) The calibration curve of absorbance at 350 nm and the concentration of azobenzene. Data were presented as mean ± s.d (n=3). (d) The thermogravimetric analysis (TGA) curves of the functionalized nanoparticles.

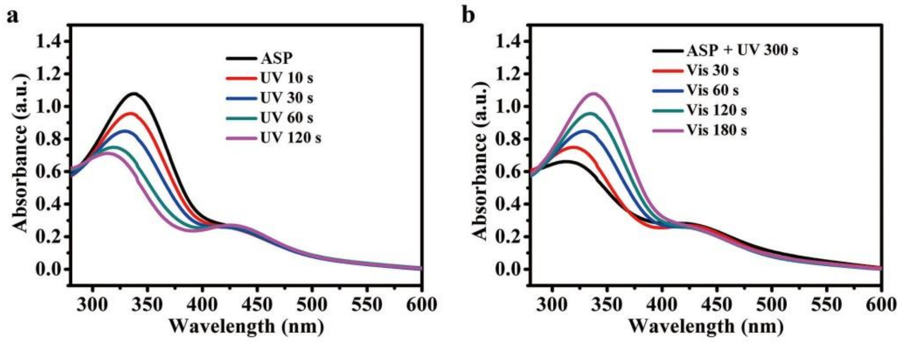

Supplementary Figure 15. The UV-Vis spectra of ASP under UV (a) or Vis (b) light. The changes of spectra indicated that the Azo modified on the SP still retained the photoresponse activity.

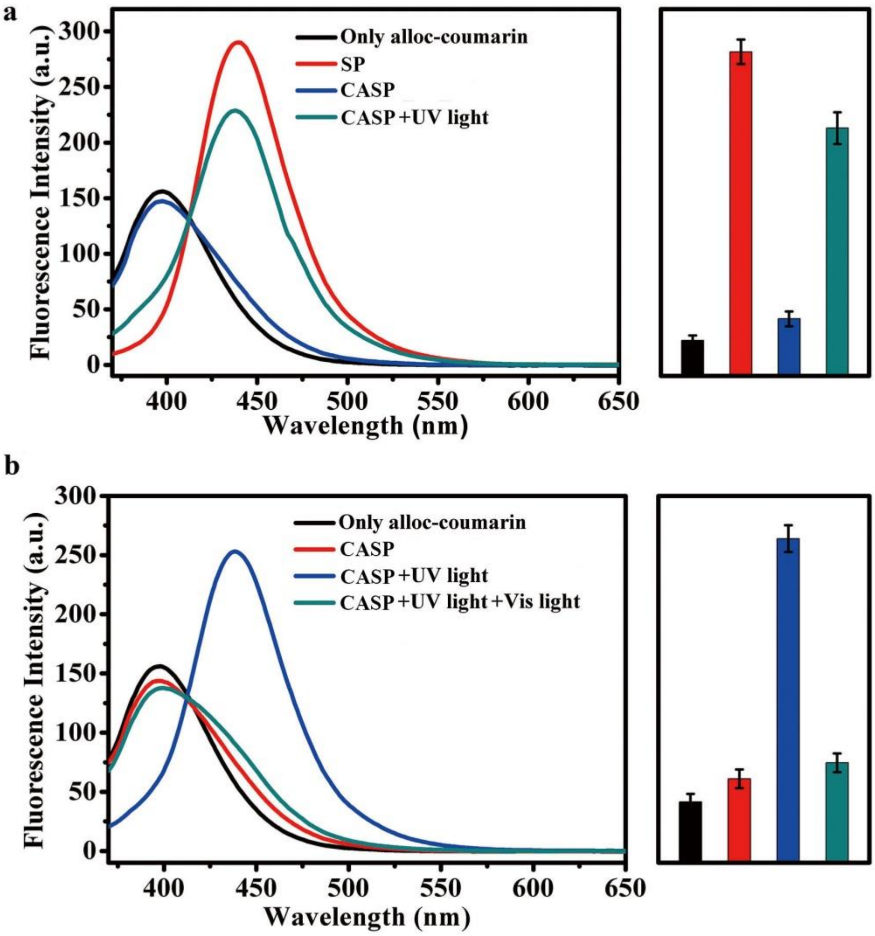

Supplementary Figure 16. The fluorescence spectra demonstrated the light activation processes on SP and CASP. (a) The fluorescence spectra of alloc-coumarin alone (black line), alloc-coumarin under SP (red line), alloc-coumarin under CASP (blue line), alloc-coumarin under CASP with UV light (cyan line). (b) The fluorescence spectra of alloc-coumarin alone (black line), alloc-coumarin under CASP (red line), alloc-coumarin under CASP with UV light (blue line), alloc-coumarin under CASP with UV light and Vis light (cyan line). Data were presented as mean ± s.d (n=3).

Kinetics studies of SP and CASP before and after UV light irradiation

100 µg mL^{-1} SP or CASP were mixed with 40 µM N-alloc-coumarin in 400 µL DI water. The fluorescence intensity was recorded continuously by JASCO F-6000

fluorescence spectrometer under the time-course model (Ex / Em = 360 / 450 nm). 40 minutes later, we irradiated the mixtures by UV light for 10 min and the reaction process was monitored for another 40 min. The fluorescence changes of CASP indicated that UV light could reactivate the catalytic activity of CASP though the isomerization of azobenzene.

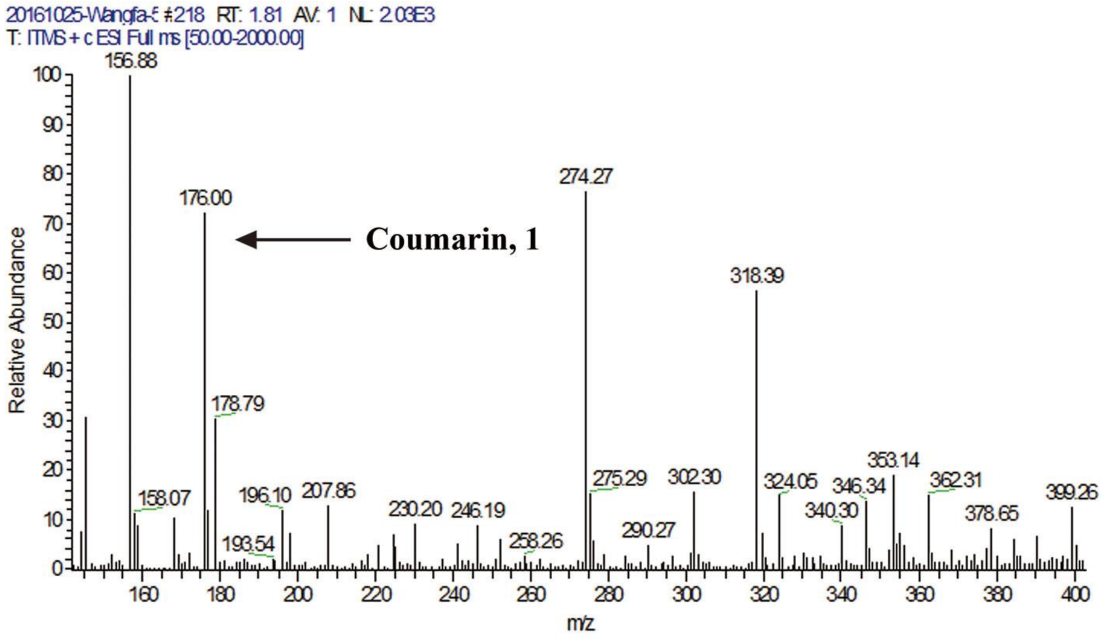

Supplementary Figure 17. LC-MS analysis comfirming the prescence of coumarin, 1, after the catalytic reaction of CASP with UV light.

Recyclable test of SP and CASP

The nanocatalysts were collected from the solution after the catalytic reaction had occurred. For CASP, the solution was irradiated by Vis light for 20 min before centrifugate. The precipitates were washed with water (2 mM CD solution for CASP) for 3 times. Then the recycled nanoparticles were diluted with water, PBS and DMEM medium again for next round experiments. The slight reduced yield of the recycled catalysts was due to the loss of catalysts in centrifugal processes.

Supplementary Table 3 Recyclable test of nanocatalysts

entry	solvent	N-alloc-coumarin	Recycled SP	Recycled CASP With UV light	Yield of 2
1	water	+	-	-	0
2	PBS	+	-	-	0
3	DMEM	+	-	-	0
4	water	+	+	-	85
5	PBS	+	+	-	85
6	DMEM	+	+	-	82
7	water	+	-	+	81
8	PBS	+	-	+	80
9	DMEM	+	-	+	78
10	water	+	-	+*	6
11	PBS	+	-	+*	4
12	DMEM	+	-	+*	3

In the groups labeled with *, the recycled CASP was not treated with UV light.

Supplementary Note 3.

Cellular uptake of the nanocatalysts tracked by ICP-MS

Nanocatalysts SP and CASP (40 µg mL^{-1}) were incubated with pre-seeded HeLa cells in 24-well plates (around 20,000 cells per well). After 24 h, old media were removed and cells were washed three times with PBS (500 µL). Then lysis buffer (300 µL) was added to the cells. The resulting cell lysate was digested overnight using HNO_3 and H_2O_2 (3:1 ml). On the following day, aqua regia (3 mL) was added, and then the sample was allowed to react for another 2 - 3 h. The sample solution was then diluted to 10 mL with deionized water and aqua regia with the final solution containing 5%

aqua regia. Cellular uptake experiments with the different nanoparticles were repeated in triplicate, and the each replicate was measured 3 times by ICP-MS.

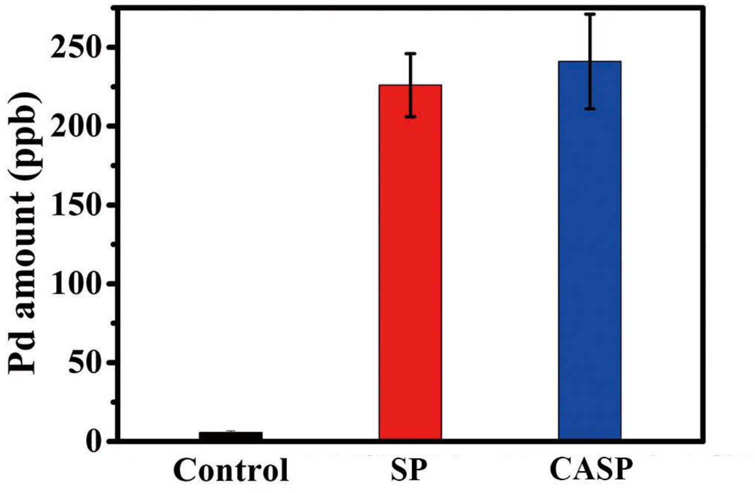

Supplementary Figure 18. The cellular uptake of SP and CASP were tracked by ICP-MS. Data were presented as mean ± s.d (n=3).

Cell viability assay

Toxicity of nanocatalysts were checked using MTT assay. HeLa cells were grown in a cell culture flask using low-glucose Dulbecco’s modified Eagle medium (DMEM) supplemented with 10% fetal bovine serum at 37 °C in a humidified atmosphere of 5% CO_2. When cells were more than 80% confluent, HeLa cells were seeded at 10,000 cells per well in 96-well plates 24 h prior to the experiment. During the experiment, old media were removed and cells were washed with 100 µL PBS once. Then, cells were treated in triplicate with 100 µL of nanoparticles with different concentrations (10, 20, 40, 60, 80, 100, 150 µg mL^{-1}). After 24 h cells were washed with 100 µL PBS three times and treated with 100 µL of MTT solution in DMEM (10 % solution) for 4 h. Subsequently, the supernatant was discarded, followed by the additional of 100 mL DMSO into each well and incubation in the shaker incubator with gentle shakes. Then the optical density (OD) was read at a wavelength of 490 nm. Results demonstrated that both ASP and CASP showed high cell viability.

Additionally, the biocompatibility of low dose UV light and Vis light were also comfirmed in this study. The incubated cells were exposed to 365 nm UV light (0.12 W cm^{-2}) or Vis light for 0, 1, 2, 4, 6, 10 min. After 24 h, the cells were washed with 100 μL PBS three times and treated with MTT solution for 4 h. Subsequently, the optical density (OD) was read at a wavelength of 490 nm as mentioned above.

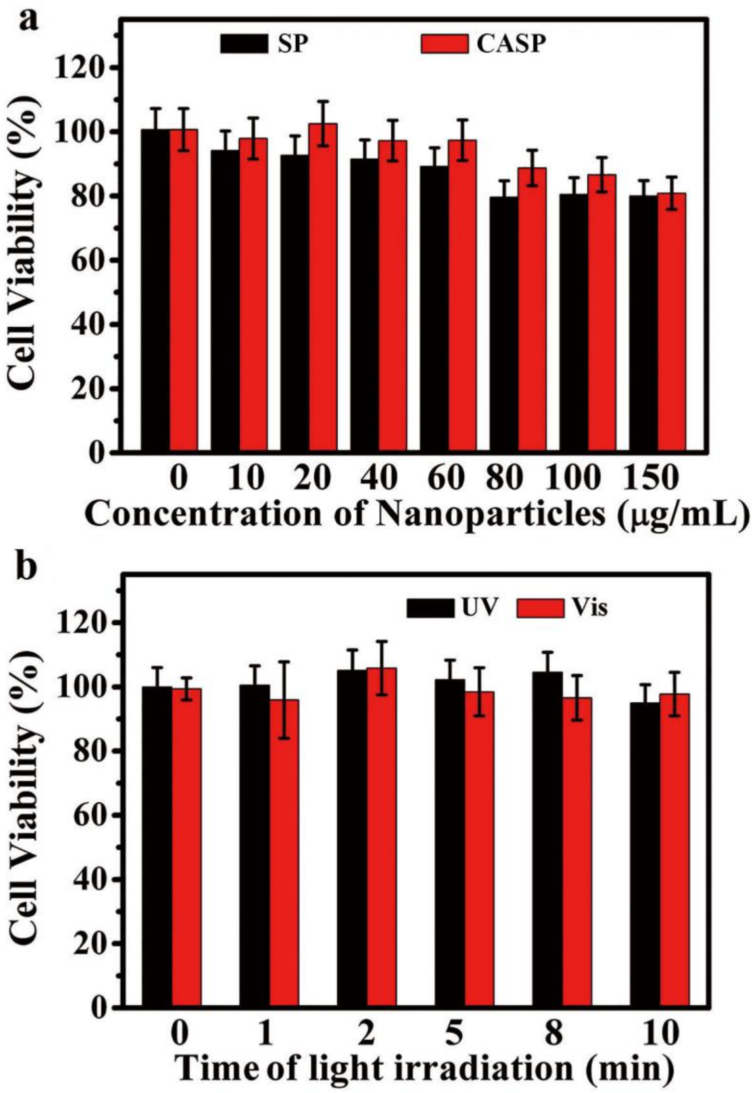

Supplementary Figure 19. (a) The biocompatibility of SP and CASP with different concentrations on HeLa cells by MTT assay. (b) The MTT of UV light (0.12 W cm^{-2}) and Vis light irradiation with different time on HeLa cells. Data were presented as mean ± s.d (n=3).

SP induced allylcarbamate cleavage inside living cells

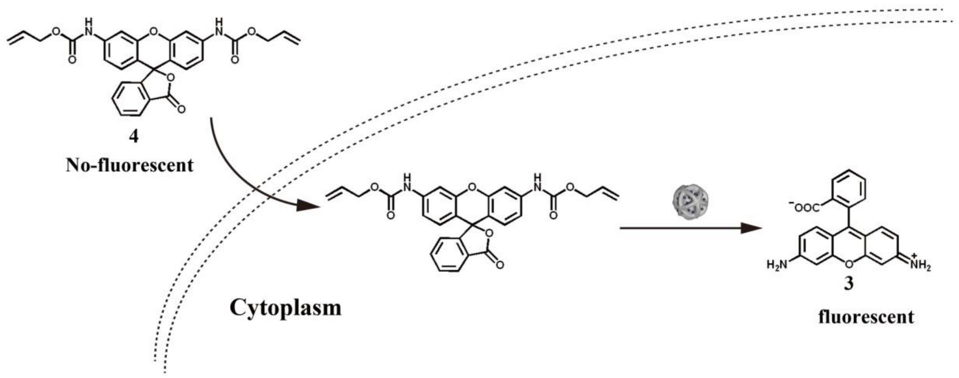

Experiments analysed by flow cytometry HeLa cells were plated in 6-well paltes. Nanocatalysts SP or CASP (40 µg mL^{-1}) were incubated with pre-seeded HeLa cells. After 24 h incubation, old media were removed and cells were washed three times with PBS to eliminate extracellular nanoparticles. Protected Rhodamine 110, **4** (10 mM in DMSO) was added to a final concentration of 20 µM and incubated for 24 h. After that, cells were washed with PBS twice, harvested with trypsin and resuspended in PBS buffer. The intracellular prescence of fluorescent compound **3** was analyzed by flow cytometry under FITC-like band pass emission filters (530/30).

Experiments analysed by fluorescence image For this study, HeLa cells were cultured on sterilised cover slips in 24-well plates overnight. SP was incubated with pre-seeded HeLa cells. After 24 h, old media were removed and cells were washed three times with PBS to eliminate extracellular nanoparticles. Protected Rhodamine 110, **4** (10 mM in DMSO) was added to a final concentration of 20 µM and incubated for 24 h. After that, cells were washed with PBS twice, the nuclei were stained by incubation with a 10 µg mL^{-1} solution of Hoechst 33258 in media for 15 min. The fluorescence images were collected by OLYMPUS-BX51 microscopes with U-25ND25 filter.

The light activation of CASP inside living cells

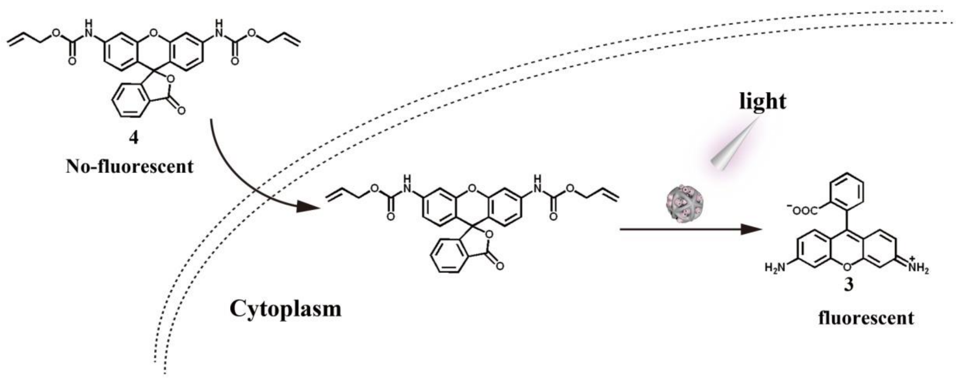

We incubated CASP or SP (40 μg mL^{-1}) with in 6-well or 24-wells paltes for 24 h. old media were removed and cells were washed three times with PBS to eliminate extracellular nanoparticles. Then, the cells were treated with 0.12 W/cm^2 UV light for 10 min. Protected Rhodamine 110, **4** (10 mM in DMSO) was added to a final concentration of 20 μM. After incubated 24 h, cells in 6-well plates were washed with PBS twice, harvested with trypsin and resuspended in PBS buffer. The intracellular prescence of fluorescent compound **3** was analyzed by flow cytometry using FITC-like band pass emission filters (530/30). The cells in 24-well plates were washed with PBS twice, the nuclei were stained by incubation with a 10 μg mL^{-1} solution of Hoechst 33258 in media for 15 min. The fluorescence images were collected.

The experiments about intracellular reversible process in short term: HeLa cells were plated in 24-well paltes, and the catalyst CASP and non-fluorescent precursors alloc-Rhodamine 110 were incubated with the cells for 8 hours. Then, the cells were cyclically treated with UV and Vis light, the changes of fluorescence intensity were monitored with time. We carried out the experiment as following: recorded the fluorescence intensity at first 15 min, treated the cells with UV light for 10 min, recorded the fluorescence intensity for another 15 min, treated the cells with Vis light for 20 min, recorded the fluorescence intensity for 15 min. The similar processes were repeated five times. The experimental results were added in the supporting information (Supplementary Fig. S21). The faster increase of fluorescence intensity treated by UV indicated the catalytic reaction was activated and the lower increase of fluorescence intensity treated by UV indicated the catalytic reaction was inactivated

again. The results suggested that in short term our system could realize the reversible activation of TMCs in cells more than 5 cycles.

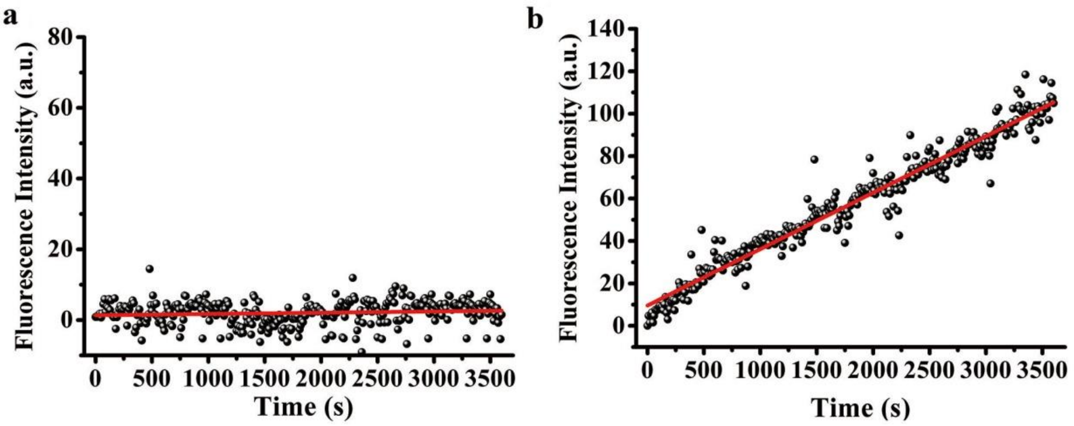

Supplementary Figure 20. The fluorescence intensity of alloc-Rhodamine 110 in cells under time course model at first 1 h. The difference between (a) CASP and (b) CASP + UV light indicated the UV light could activate the catalyst CASP effectively.

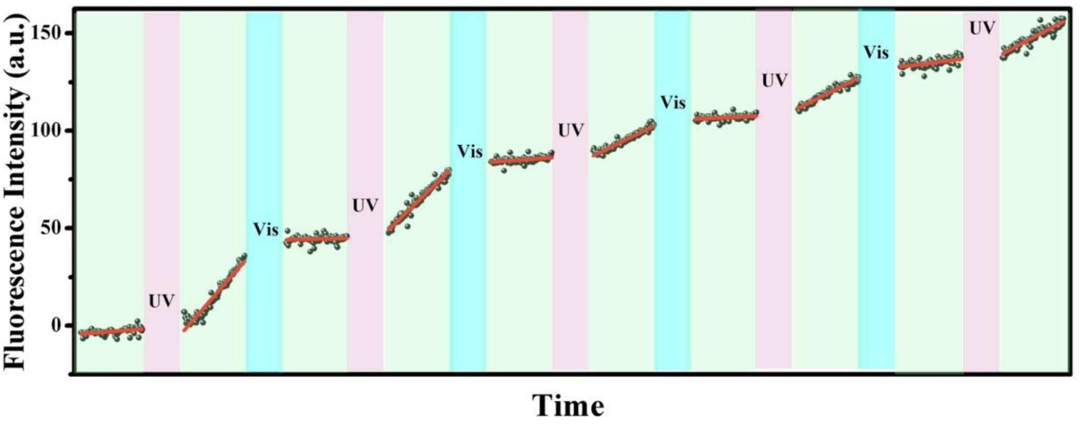

Supplementary Figure 21. The experiment about reversible processes in cells of our system. The fluorescence intensity of alloc-Rhodamine 110 in cells dealt with UV or Vis light under time course model. The results suggested that in short term our system could effectively realize the reversible activation of TMCs in cells more than 5 cycles.

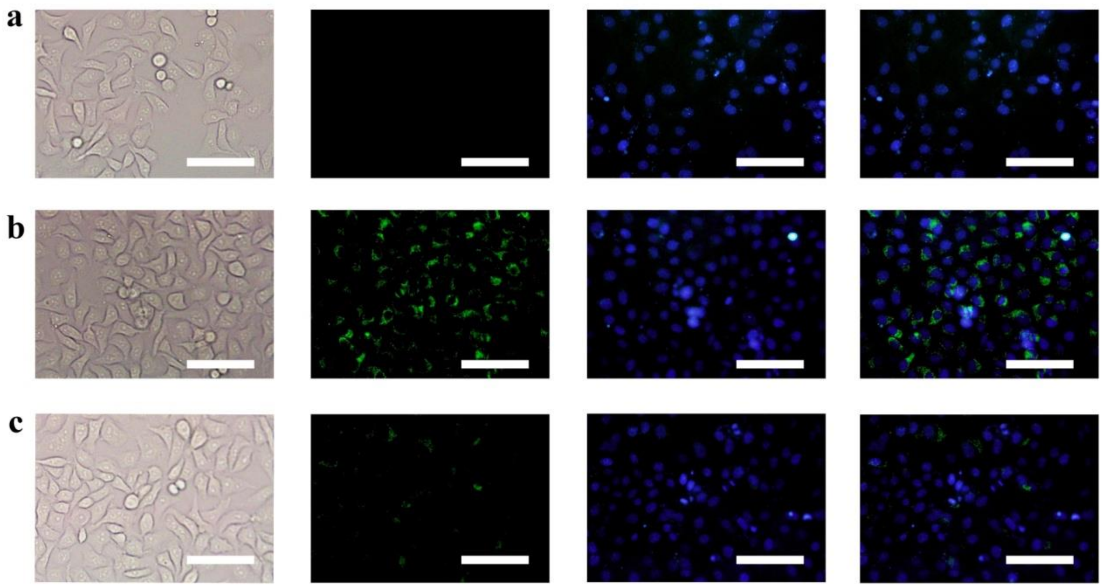

Supplementary Figure 22. The fluorescent images of CASP. (a) control, (b) CASP with UV light, (c) CASP with UV light and Vis light. (Scale bar = 80 μm)

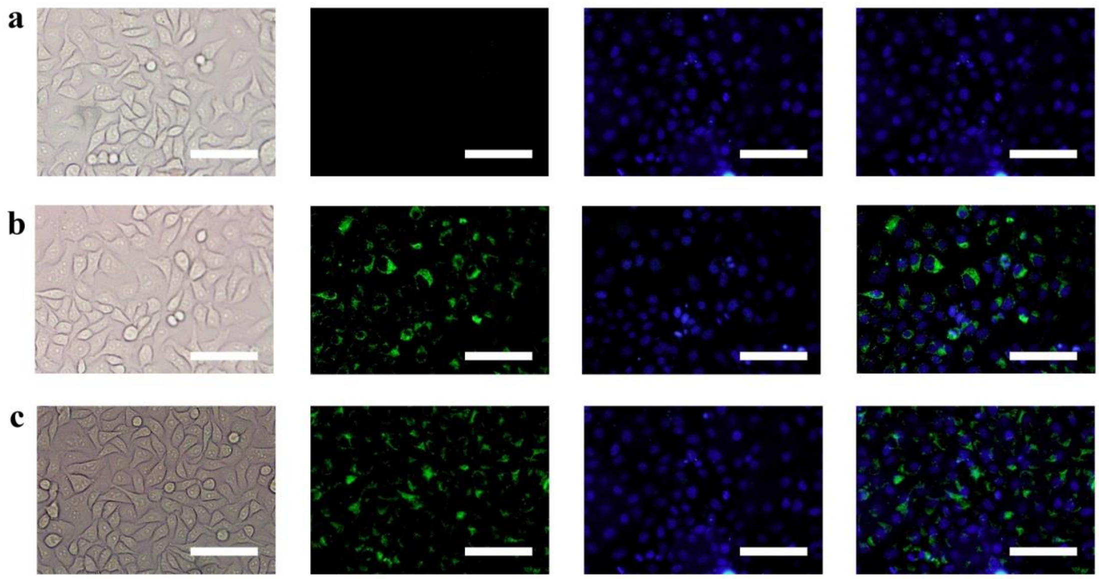

Supplementary Figure 23. The fluorescent images of SP. (a) control, (b) SP with UV light, (c) SP with UV light and Vis light. (Scale bars =80 μm)

LC-MS study of light-gated allylcarbamate cleavage in HeLa cells

The reaction was carried out in HeLa cells in 6-well plates under the same conditions as described in flow cytometry experiments. After the reaction, the cells were washed extensively with PBS. The cells were detached with trypsin, harvested with water and lysed by sonication and the lysate was centrifuged for 5 min at 13000 rpm. The supernatant was collected and mixed with cold acetone, under -20 °C overnight. The mixtures was centrifuged for 15 min at 13000 rpm. The solvent was removed under vacuum and the residue was dissolved in methanol and analyzed by LC-MS.

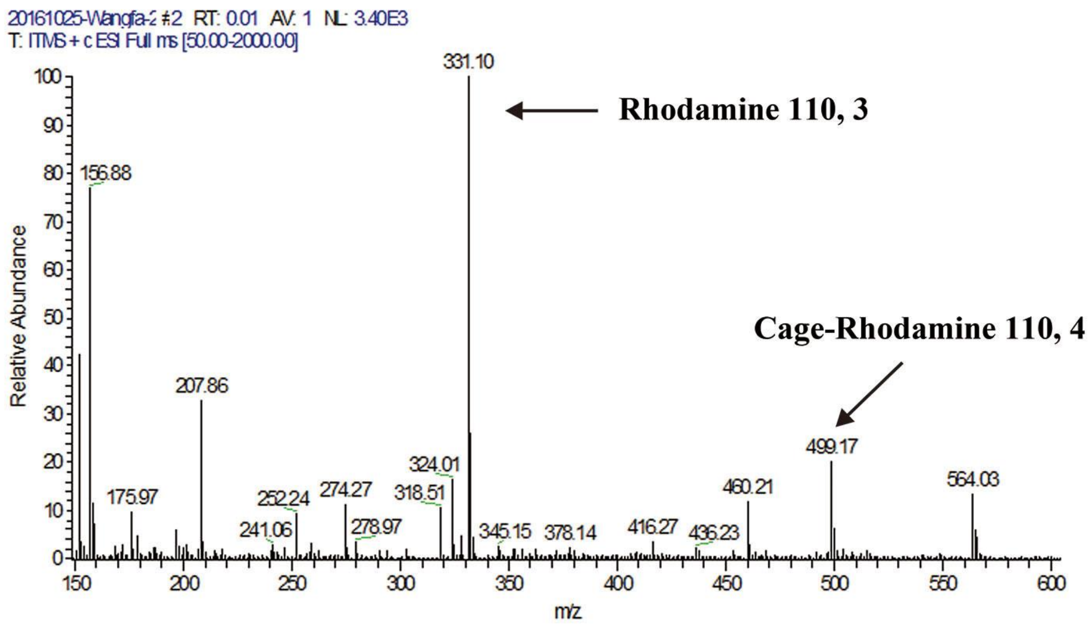

Supplementary Figure 24. LC-MS analysis of the cell lysate under CASP with UV light, confirming the presence of Rhodamine 110, **3**.

Supplementary Note 4.

CASP mediated Suzuki-Miyaura Coupling Reaction

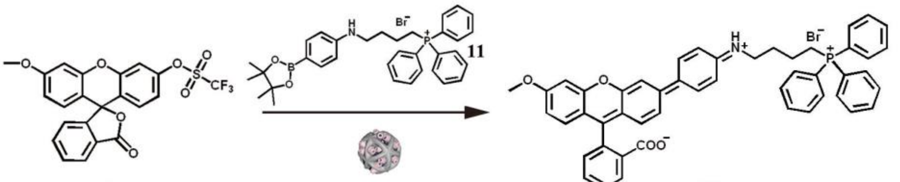

HeLa cells were incubated on sterilized cover slips in a 24-wells plate (around 5,000 cells per well) at 37 °C for 24 h. Old media were removed and replaced by fresh media containing SP or CASP (40 µg mL^{-1}) and incubated for another 24 h. Excess of extracellular nanoparticles were removed by washing with PBS for three times. Compounds **8** and **11** (20 mM in DMSO) were diluted in fresh media to give a final concentration of 20 µM and incubated at 37 °C for another 24 h. After that, the cells were washed with PBS twice, Subsequently, mitochondria were stained for 20 min by 50 nM of Mito Tracker® Red CMXRos at 37 °C. The nuclei were stained for 15 min by a solution of Hoechst 33258 (10 µg mL^{-1}). The fluorescence images were collected.

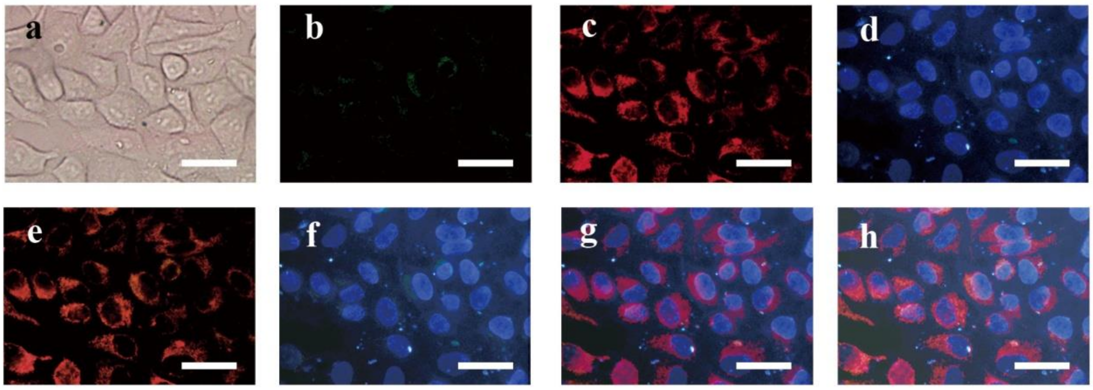

Supplementary Figure 25. The fluorescence images of the CASP catalyzing the Suzuki-Miyaura Coupling Reaction without the UV light, the low fluorescence intensity of green channel indicated the low catalytic activity of CASP as we designed. (a) bright field image of HeLa cells, (b) emission of the compound 12 (Green channel), (c) the mitochondrion labeled with Mito Tracker® Red CMXRos (Red channel), (d) the cell nucleus labeled with Hoechst 33258 (Blue channel), (e) merging with b and c, (f) merging with b and d, (g) merging with c and d, (h) merging with b, c and d. (Scale bar = 10 µm)

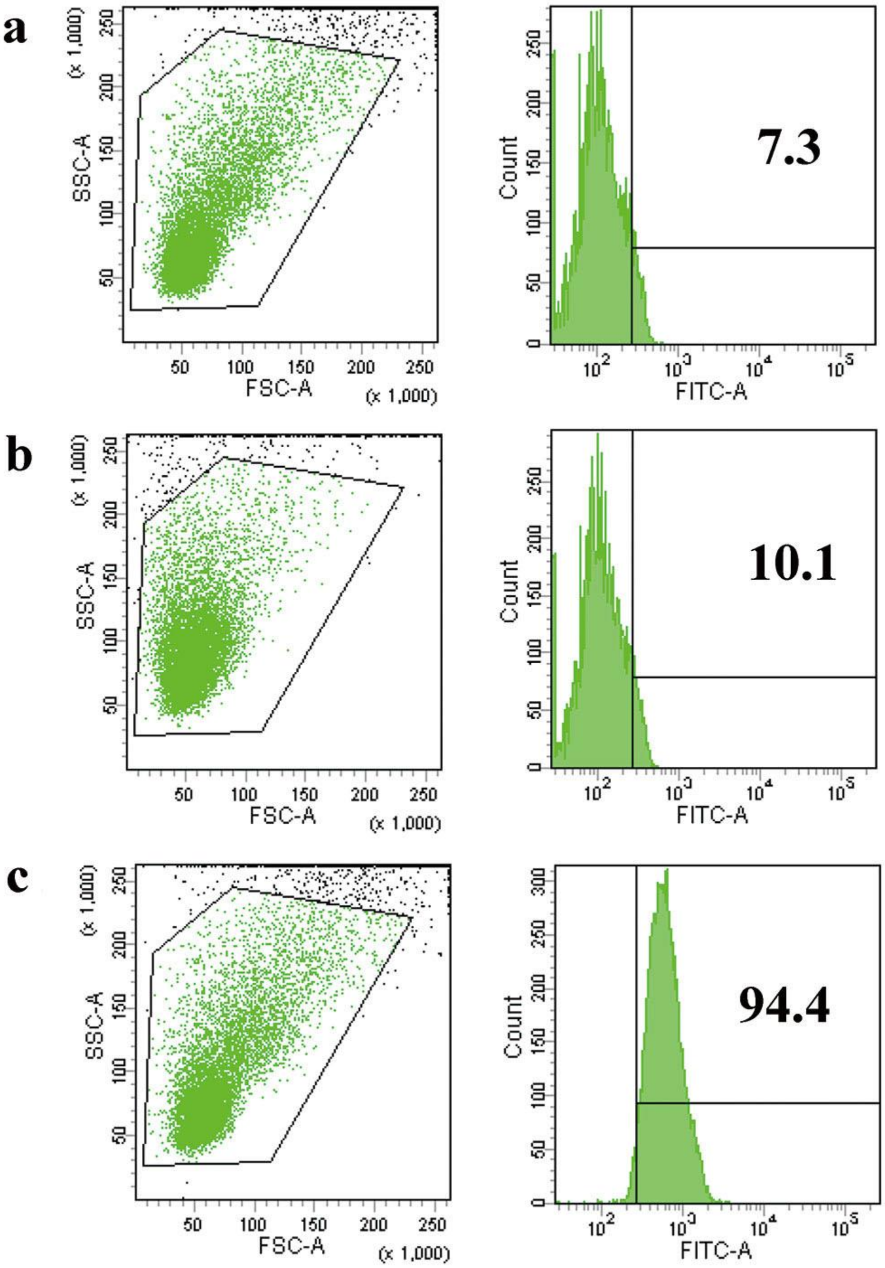

Supplementary Figure 26. The flow cytometry analysis of HeLa cell showing intracellular synthesis of dye **12**. (a) Control: untreated HeLa cells. (b) The HeLa cells treated with CASP without UV light. (c) The HeLa cells treated with CASP with UV light.

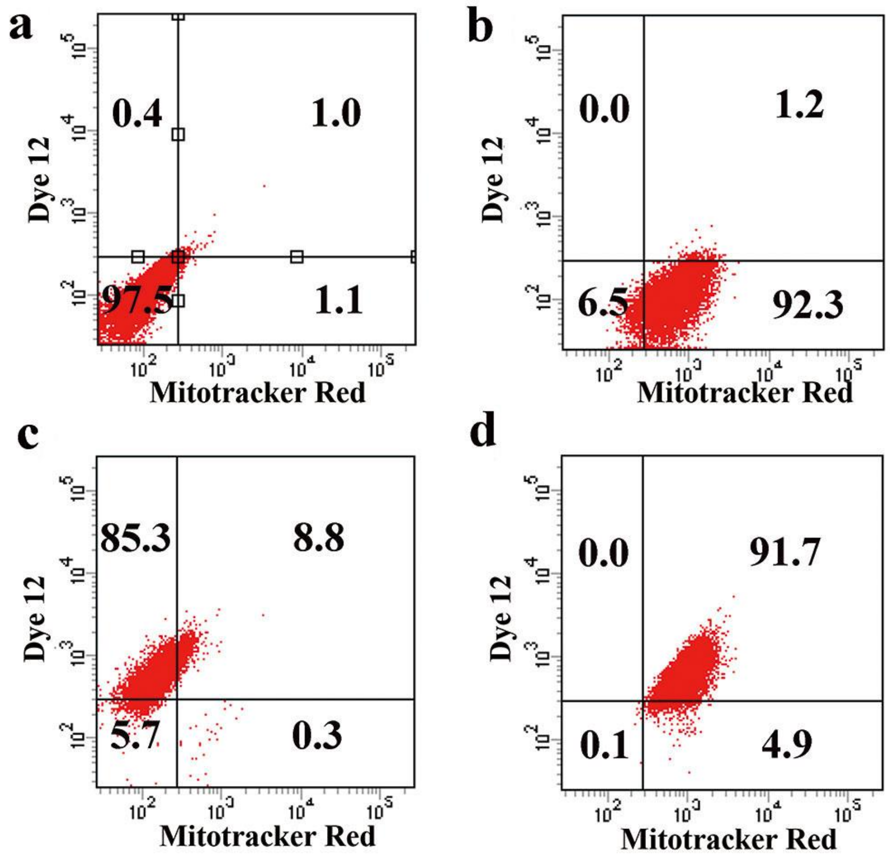

Supplementary Figure 27. The two-channel flow cytometry analysis of HeLa cells verified the independence between dye **12** and MitoTracker® Red CMXRos for probing the cells. (a) Control HeLa cells treated with nothing. (b) The cells labeled with MitoTracker® Red CMXRos. (c) The cells treated with CASP and UV light. (d) The cells treated both with catalysts and MitoTracker® Red CMXRos.

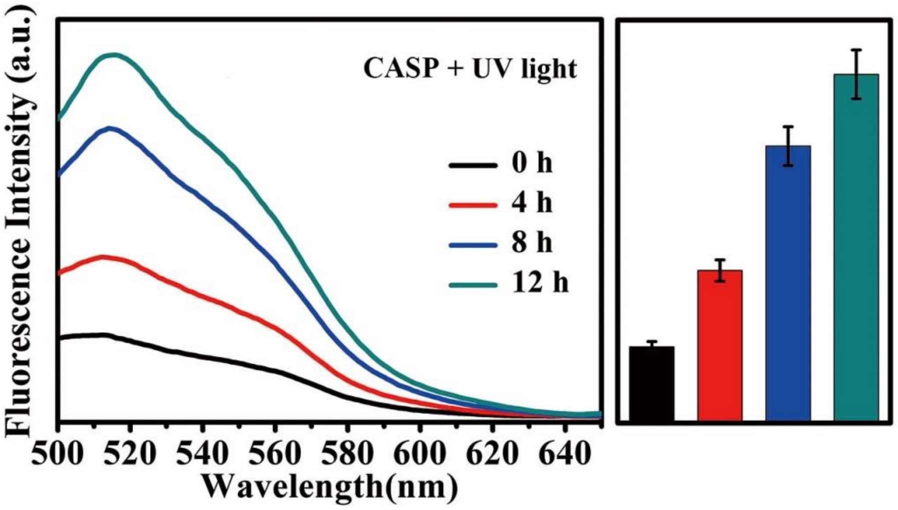

Supplementary Figure 28. The fluorescence spectra of Suzuki-Miyaura Coupling Reaction treated by CASP activated by UV light. Data were presented as mean ± s.d (n=3).

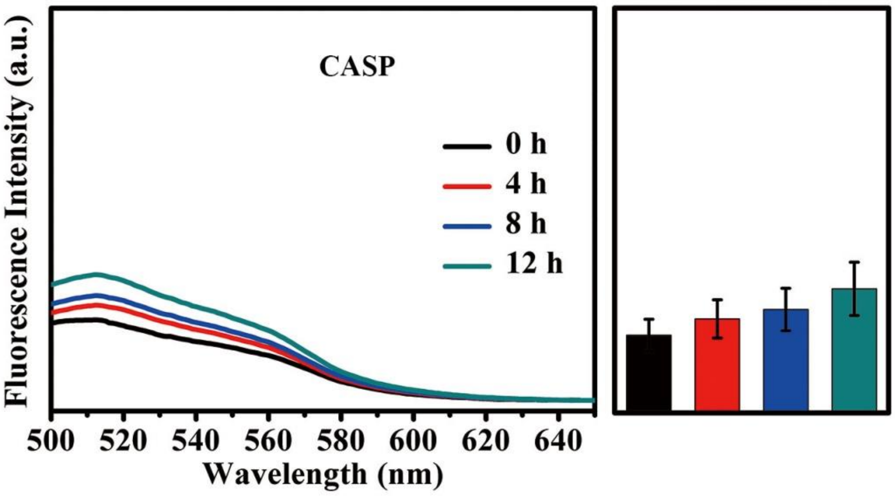

Supplementary Figure 29. The fluorescence spectra of Suzuki-Miyaura Coupling Reaction treated by CASP only. Data were presented as mean ± s.d (n=3).

LC-MS study of Suzuki-Miyaura Reaction in HeLa cells

The reaction was carried out on the HeLa cells in 6-well plates using the same conditions as described in flow cytometry experiments. After the reaction, the cells were washed extensively with PBS. The cells were detached with trypsin, harvested with water and lysed by sonication and the lysate was centrifuged for 5 min at 13000 rpm. The supernatant was collected and mixed with cold acetone, under -20 °C overnight. The mixtures was centrifuged for 15 min at 13000 rpm. The solvent was removed under vacuum and the residue was dissolved in methanol and analysed by LC-MS.

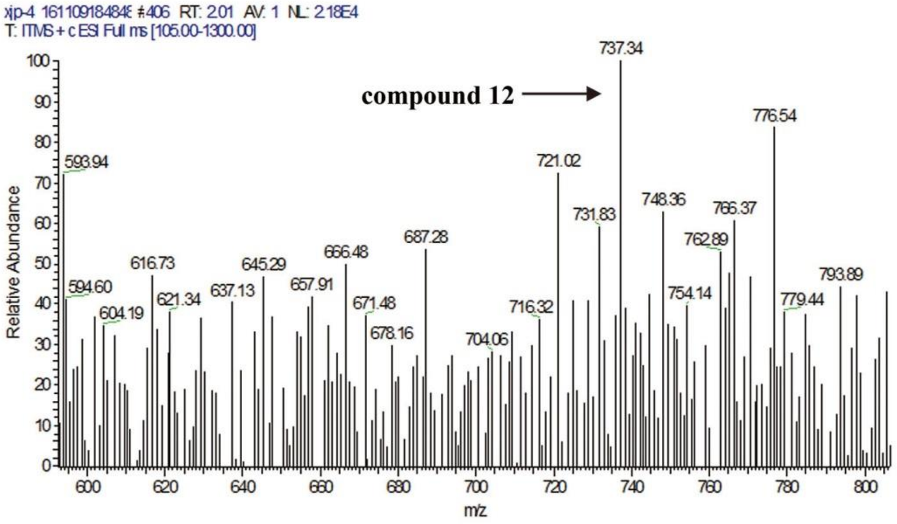

Supplementary Figure 30. The LC-MS analysis from the cell lysate confirming the presence of Suzuki-Miyaura coupling product, **12**.

Supplementary Note 5.

HeLa cells were seeded at 10,000 cells per well in 96-well plates 24 h prior to the experiment. During the experiment, old media were removed and cells were washed with 100 µL PBS once. Then, cells were treated in triplicate with CASP (40 µg mL^{-1})

for 24 h. After mutiple washing, the cells were then incubated with different concentrations of Pro-5FU (0, 1, 5, 10, 50, 100 and 500 μM), while some of the cells were treated with Pro-5FU and UV light at the same time. After 24 h, the cell viability was evaluated by MTT analysis.

The toxicity profiles of 5FU and Pro-5FU were also investigated at different concentrations by performing a cell viability assay. The toxicity profiles of 5FU and Pro-5FU were investigated by performing a cell viability assay (Figure S31b). Cells were treated with 5FU or Pro-5FU at various concentrations from 10 nM to 1 mM. Although 5FU showed toxicity as its concentration increased, Pro-5FU retained a high cellular viability at all concentrations studied.

As shown in Figure S31c, cells that were incubated with CASP + UV light showed elevated toxicity at a higher concentration of Pro-5FU, while CASP alone retained ~100% cell viability even at higher concentrations of Pro-5FU. As expected, Pro-5FU was not toxic at any concentrations used. These results indicating that the toxicity was coming from the intracellular conversion of Pro-5FU into 5FU by gated-catalysis, but not the catalyst itself.

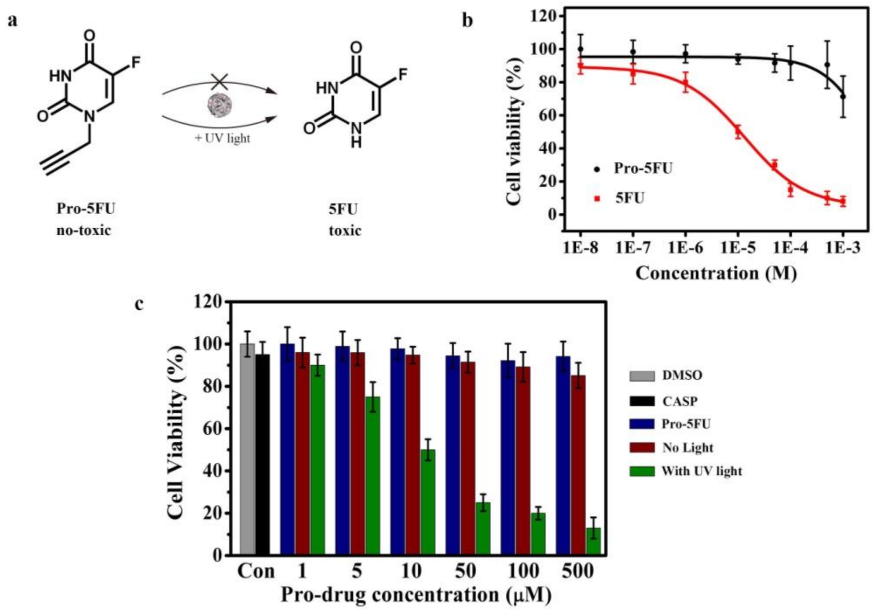

Supplementary Figure 31. (a) Structures of Pro-5FU, 5FU and the catalyst used for prodrug light-activation. (b) The toxicity profiles of 5FU and Pro-5FU at various concentrations. (c) The experiments about the light-activation of pro-drug by CASP. DMSO, CASP and Pro-5FU showed negligible cytotoxicity; CASP with UV light could

activate the intracellular conversion of Pro-5FU into 5FU, significantly reducing cell viability. Data were presented as mean ± s.d (n=3).

Coumarin fluorescence standard curve

We can estimate the concentration of the product after catalytic reaction, using the fluorescence intesnsity coming from the coumarin product. For this purpose, a fluorescence standard curve for coumarin was obtained. The stock solution of coumarin (0.1 mM) was prepared in water. Successive dilution was done, and the fluorescence intensity of prepared solutions were directly measured by fluorescence spectrometer.

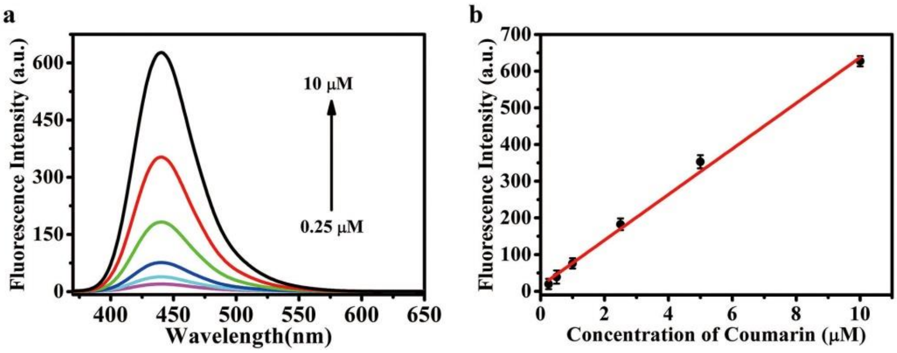

Supplementary Figure 32. The standard curve of coumarin. Data were presented as mean ± s.d (n=3).

Rhodamine 110 fluorescence standard curve

The fluorescence standard curve for rhodamine 110 was also obtained as described above.

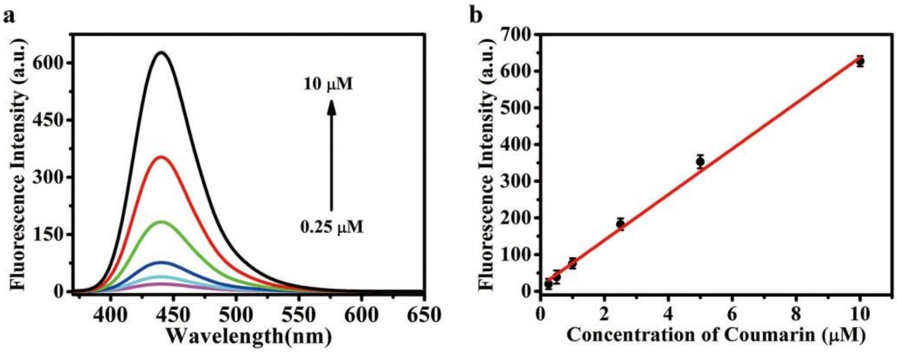

Supplementary Figure 33. The standard curve of Rhodamine 110. Data were presented as mean ± s.d (n=3).

Supplementary Note 6.

Preparation of azobenzene-COOH (Azo-COOH)

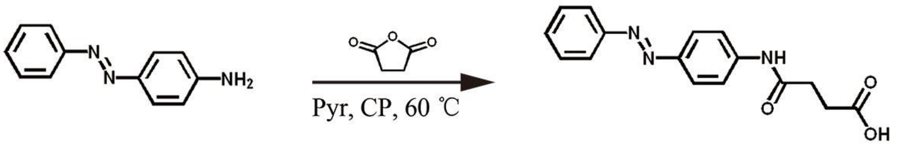

For our design, we synthesized the light switch molecule first. The (E)-4-oxo-4-((4-(phenyldiazenyl)phenyl)amino)butanoic acid (Azo-COOH) was prepared according to the literature[4]. In brief, 4-Aminoazobene (1.97 g, 10 mmol) and succinic anhydride (1.20 g, 12 mmol) were dissolved into 25 ml distilled acetone. 0.79 g (10 mmol) anhydrous pyridine was added into the solution and the mixture was stirred for 6 h at 60 ° C. The obtained suspension was filtered and after been dried at 50 ° C for 48 h under vacuum drying oven, Azobenzene-COOH was obtained. (yield: 97 %). Figure S32. shows the ^{1}H NMR spectrum of Azo-COOH.

^{1}H NMR (600 MHz, DMSO) δ 12.15 (s, 1H), 10.33 (s, 1H), 7.97 – 7.72 (m, 6H), 7.65 – 7.46 (m, 3H), 2.59 (dt, J = 44.6, 6.6 Hz, 4H).

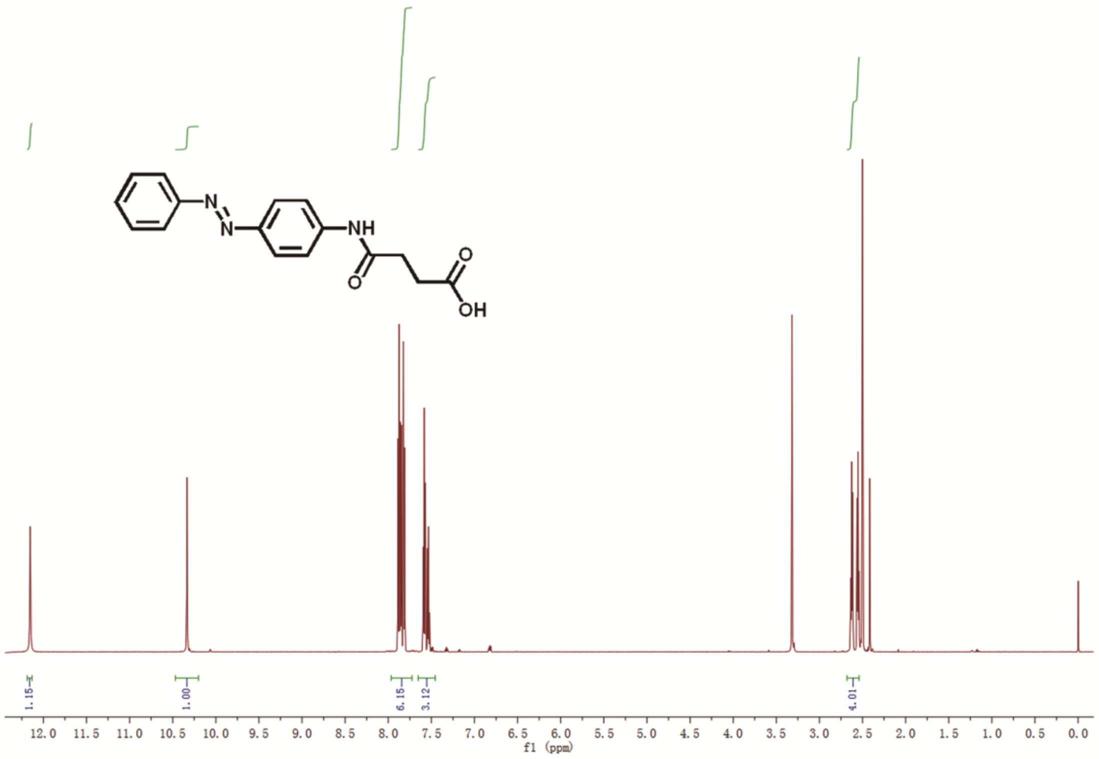

Supplementary Figure 34. The ^{1}HNMR spectrum of Azo-COOH. (H_2O δ=3.3, DMSO-d_6 δ=2.5)

Synthesis of N-allyloxycarbonyl coumarin (2)

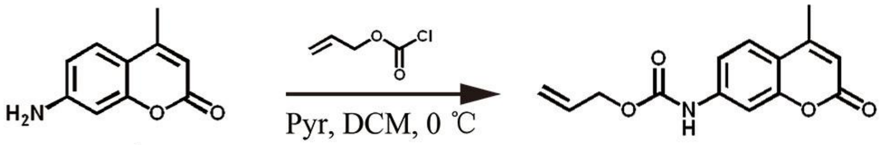

N-allyloxycarbonyl coumarin: allyl (4-methyl-2-oxo-2H-chromen-7-yl) carbamate was synthesized according to the literature with a little modification.[5] In brief, 99.2 mg 7-Amino-4-methylcoumarin, **1**, and 51.3 mg pyridine was dissolved in 3 mL DCM to obtain the yellow suspension, and then 84.1 mg allylchloroformate was added to the suspension. The mixture became bright yellow suspension after stirring at 0 ^{o}C for 12 h. Then 40 mL 0.5 M HCl was added to the mixture, after filtering, washed by diethyl ether and drying, N-alloc-coumarin was obtained, **2**, and the product was used without further purification (Yield: 87%).

^{1}H NMR (600 MHz, DMSO) δ 10.24 (s, 1H), 7.69 (d, *J* = 8.7 Hz, 1H), 7.54 (d, *J* = 1.9 Hz, 1H), 7.41 (dd, *J* = 8.7, 2.0 Hz, 1H), 6.24 (d, *J* = 1.1 Hz, 1H), 6.00 (ddd, *J* = 22.7,

10.8, 5.5 Hz, 1H), 5.32 (ddd, J = 13.8, 11.8, 1.5 Hz, 2H), 4.65 (d, J = 5.5 Hz, 2H), 2.39 (d, J = 0.9 Hz, 3H).

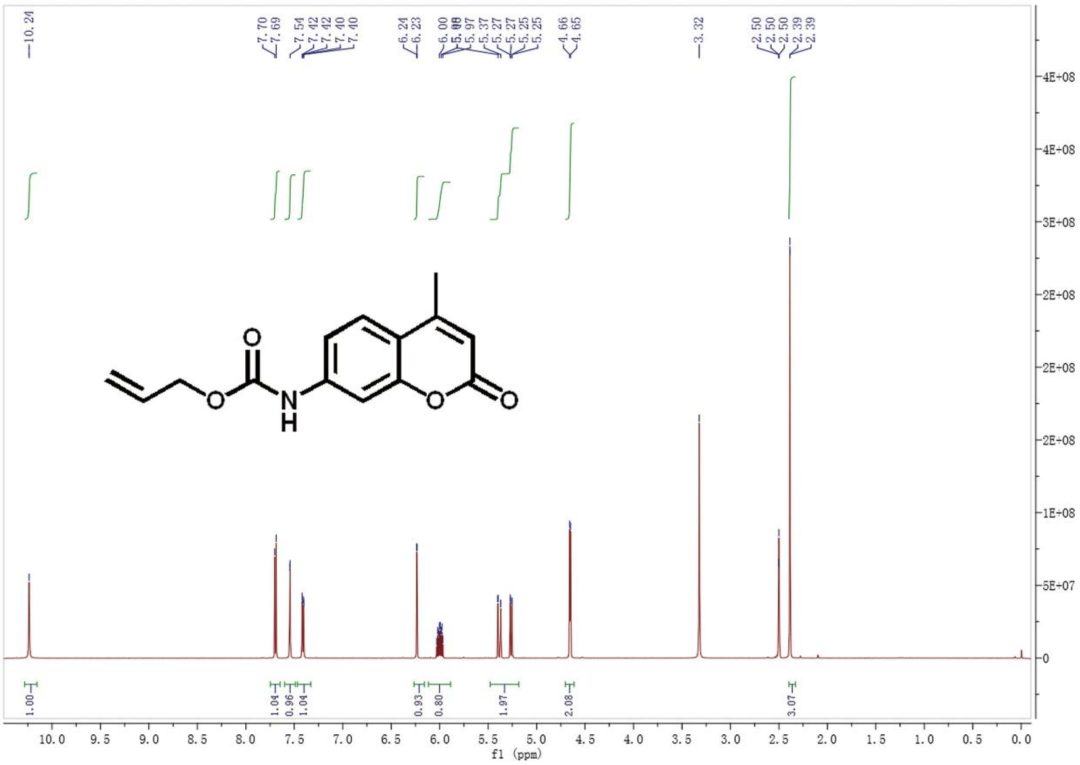

Supplementary Figure 35. The ^{1}HNMR spectrum of N-alloc-coumarin, **2**. (H_2O δ=3.3, DMSO-d_6 δ=2.5).

Synthesis of bis-allyloxycarbonyl-protected Rhodamine 110 (4)

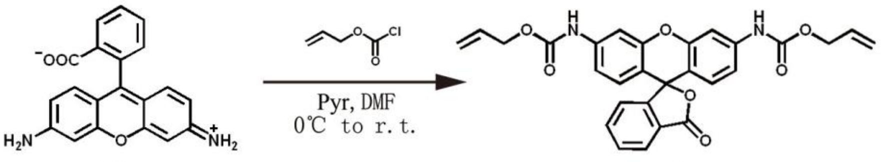

3 **4**

Bis-allyloxycarbonyl protected Rhodamine 110, **4**, was synthesised from Rhodamine 110, **3**, as previously described.[5] Briefly, Rhodamine 110 (250 mg) was dissolved in dry DMF (1.5 mL) under nitrogen and cooled to 0°C. In a separate flask, pyridine (174 µL, 2.15 mmol) was dissolved in DMF (0.6 mL). To the solution containing rhodamine 110 were added simultaneously the solutions containing pyridine and allylchloroformate (152 µL, 1.43 mmol) dropwise. The resulting red reaction mixture

was allowed to warm to room temperature while stirring overnight. Thereafter, 10 mL of EtOAc was added to the metallic red solution which was transferred to a separatory funnel and washed twice with 5% HCl (10 mL each). Small amounts of saturated $NaHCO_3$ were used to eliminate emulsion formation. The ethyl acetate layer was collected and combined with two further ethyl acetate washings (10 mL each), washed with saturated $NaHCO_3$ (10 mL), dried with anhydrous $MgSO_4$, and concentrated in vacuo. Trace amounts of DMF were removed under low pressure overnight. The resulting oil was subjected to column chromatography on a short column with hexane/ethyl acetate (2:1) as the eluting solvent. The resulting product was isolated as a white solid. ^{1}H-NMR (600 MHz, DMSO-d_6) δ (ppm) 10.07 (s, 2H), 8.00 (d, J = 7.4 Hz, 1H), 7.78 (td, J = 7.4, 1.2 Hz, 1H), 7.71 (td, J = 7.4, 1.0 Hz, 1H), 7.56 (d, J = 2.0 Hz, 2H), 7.27 (d, J = 7.5 Hz, 1H), 7.14 (dd, J = 8.7, 2.1 Hz, 2H), 6.70 (d, J = 8.7 Hz, 2H), 5.98 (m, 2H), 5.36 (ddt, J = 17.2, 1.7, 1.6 Hz, 2H), 5.24 (ddt, J = 10.4, 1.6, 1.4 Hz, 2H), 4.62(dt, J=5.5, 1.4 Hz, 2H).

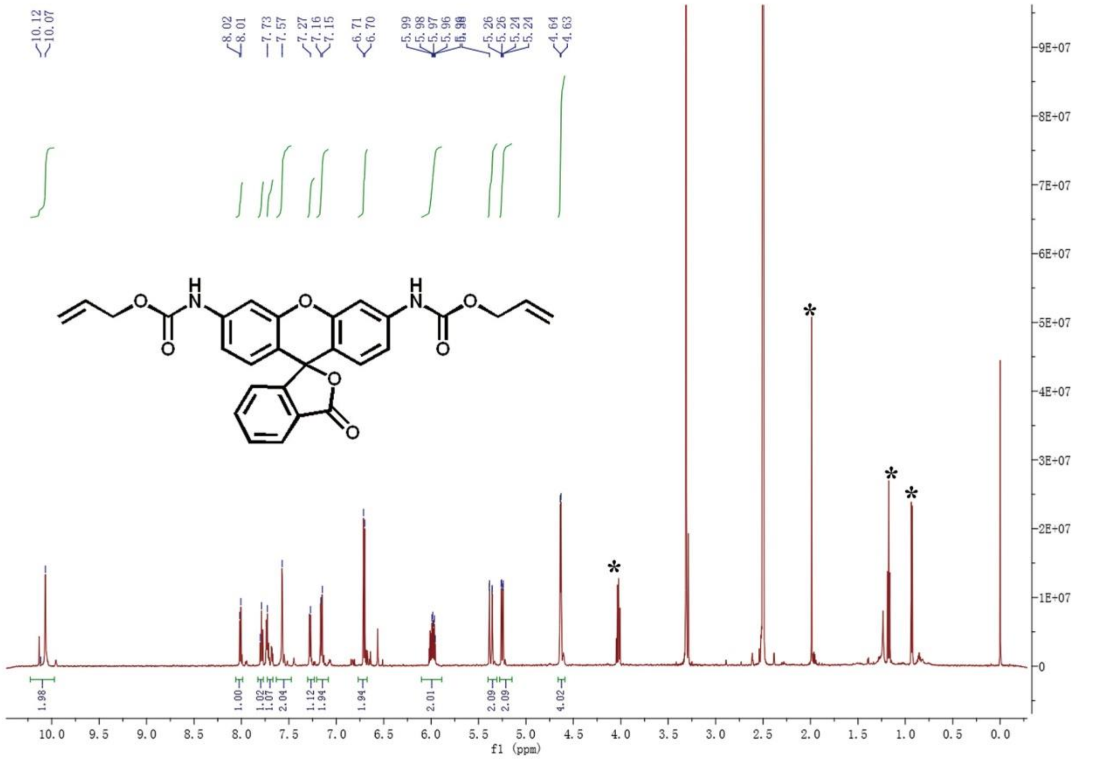

Supplementary Figure 36. The ^{1}HNMR spectrum of bis-allyloxycarbonyl protected Rhodamine 110, **4**. (H_2O δ=3.3; DMSO-d_6 δ=2.5; the peaks labeled with * were owing to ethyl acetate)

Synthesis of 3'-(trifluoromethanesulfonyl)fluorescein (7)

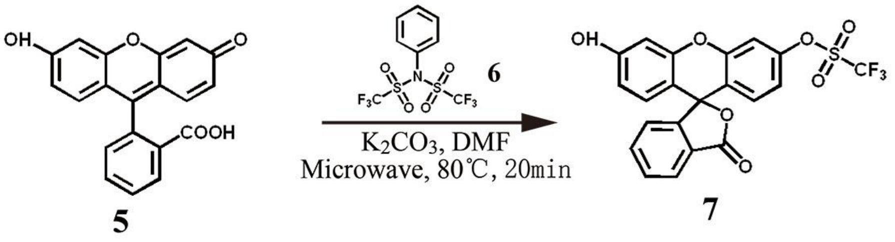

3'-(trifluoromethanesulfonyl) fluorescein, **7**, was synthesized from fluorescein, **5**, as previously described.[6] Briefly, *N*-phenyl-bis(trifluoromethanesulfonimide) (1.8 mmol) Fluoroscein, **5** (1.6 mmol), and potassium carbonate (2.4 mmol) were stirred in DMF (16 mL) and microwave-irradiated at 80 °C for 20 minutes. The reaction mixture was partitioned between EtOAc (120 mL) and 1M HCl (80 mL), the aqueous layer was extracted with EtOAc (2 x 30 mL), and the combined organics dried ($MgSO_4$) and reduced *in vacuo*. Purification of the residue via chromatography on silica gel (4:1 Hex:EtOAc) afforded an off-white solid, **7**. ^{1}H NMR (600 MHz, DMSO) δ 10.27 (s, 1H), 8.04 (d, *J* = 7.7 Hz, 1H), 7.81 (d, *J* = 7.4 Hz, 1H), 7.76 (t, *J* = 7.5 Hz, 1H), 7.70 (d, *J* = 2.5 Hz, 1H), 7.38 (d, *J* = 7.7 Hz, 1H), 7.24 (dd, *J* = 8.8, 2.5 Hz, 1H), 7.02 (d, *J* = 8.8 Hz, 1H), 6.75 (s, 1H), 6.63 (s, 2H).

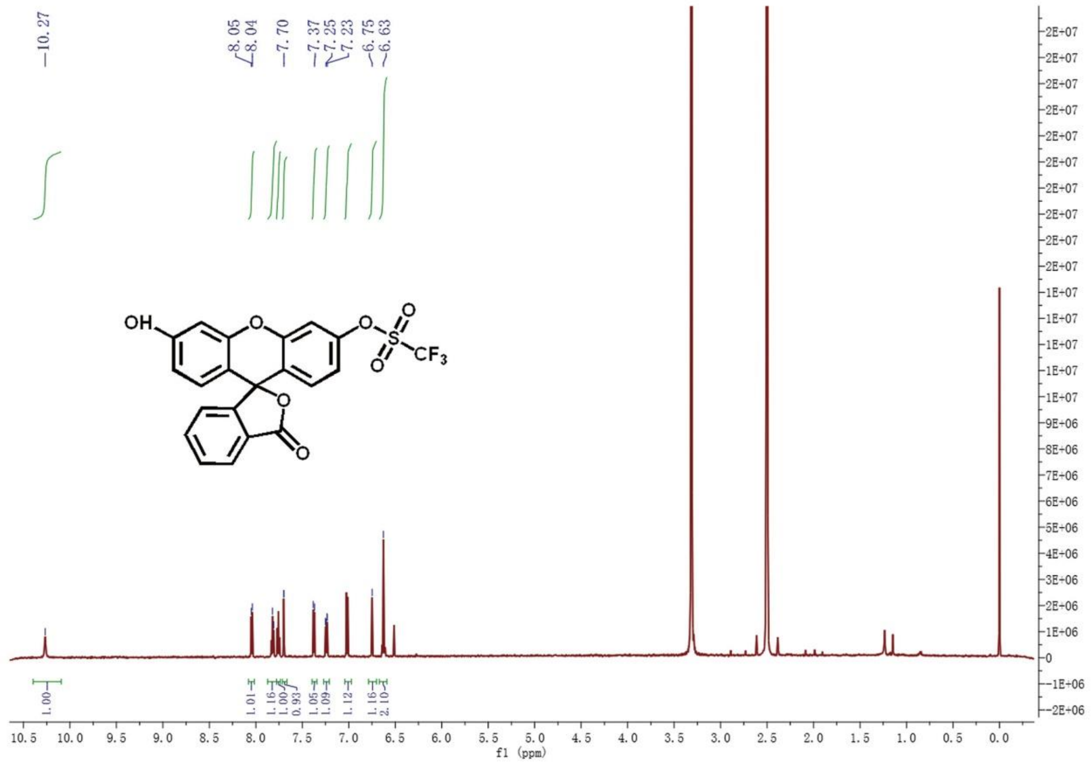

Supplementary Figure 37. The ^{1}HNMR spectrum of 3’-(trifluoromethanesulfonyl) fluorescein, **7**. (H_2O δ=3.3; DMSO-d_6 δ=2.5)

Synthesis of 6’-methyl-3’-(trifluoromethanesulfonyl)fluorescein (8)

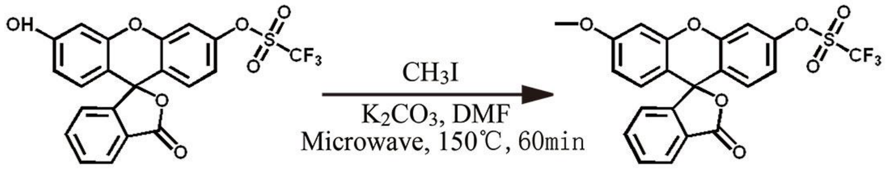

7 **8**

3’-(trifluoromethanesulfonyl)fluorescein, **7**, (0.4 mmol), methyl iodide (2.0 mmol) and potassium carbonate (0.6 mmol) were stirred in 10 mL of dry DMF and microwave-irradiated at 150 °C for 60 minutes. The mixture was partitioned between EtOAc (120 mL) and 1 M HCl (80 mL), the aqueous layer was extracted with EtOAc (2 × 30 mL), and the combined organics dried and reduced in vacuo. Purification of the residue via column chromatography on silica gel (2:1 Hexane:EtOAc) afforded compound **8** as a colourless solid. ^{1}H NMR (600 MHz, DMSO) δ 8.11 – 8.00 (m, 1H), 7.82 (s, 1H), 7.77 (d, J = 7.6 Hz, 1H), 7.70 (d, J = 2.5 Hz, 1H), 7.38 (d, J = 7.7 Hz,

1H), 7.25 (d, J = 2.5 Hz, 1H), 7.05 (d, J = 8.9 Hz, 1H), 6.99 (d, J = 2.4 Hz, 1H), 6.88 – 6.64 (m, 2H), 3.83 (d, J = 8.3 Hz, 3H).[7]

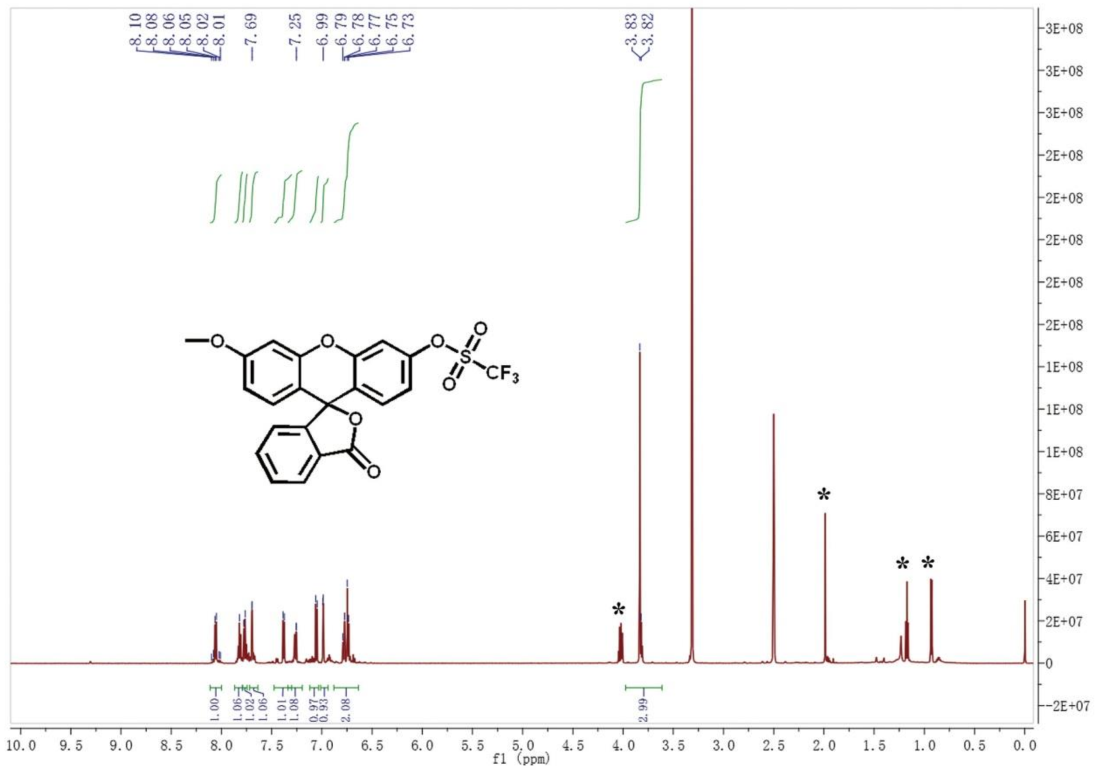

Supplementary Figure 38. ^{1}HNMR spectrum of 6'-methyl-3'-(trifluoromethanesulfonyl) fluorescein, **8**. (H_2O δ=3.3; DMSO-d_6 δ=2.5; the peaks labeled with * were owing to ethyl acetate)

Synthesis of (4-[4'-(pinacolatoboron)phenylamino]butyl)triphenyl phosphonium bromide (11)

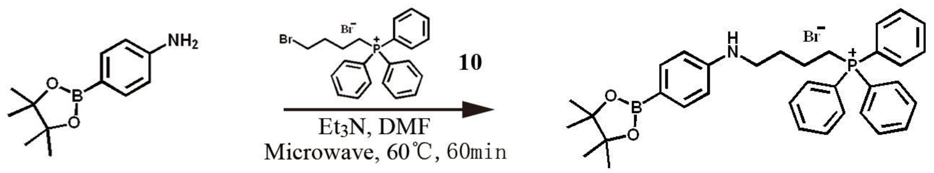

9 **11**

4-Aminophenylboronic acid pinacol ester, **9**, (0.5 mmol) and (4-bromo butyl)triphenylphosphonium bromide, 10, (0.6 mmol,) were dissolved in 10 ml DCM. Subsequently, triethylamine (2.0 mmol) was added and the reaction was microwave-irradiated for 60 min at 60 °C. The solvent was removed under reduced pressure to

give a pink solid which was purified by column chromatography on silica gel (DCM/MeOH 98:2) to yield as a colourless solid. ^{1}H NMR (600 MHz, DMSO) δ 8.05 – 7.83 (m, 7H), 7.37 (d, *J* = 8.5 Hz, 2H), 6.51 (s, 1H), 6.00 (s, 0H), 3.70 (d, *J* = 6.4 Hz, 1H), 2.04 – 1.97 (m, 1H), 1.96 – 1.82 (m, 2H), 1.81 – 1.51 (m, 6H).[7]

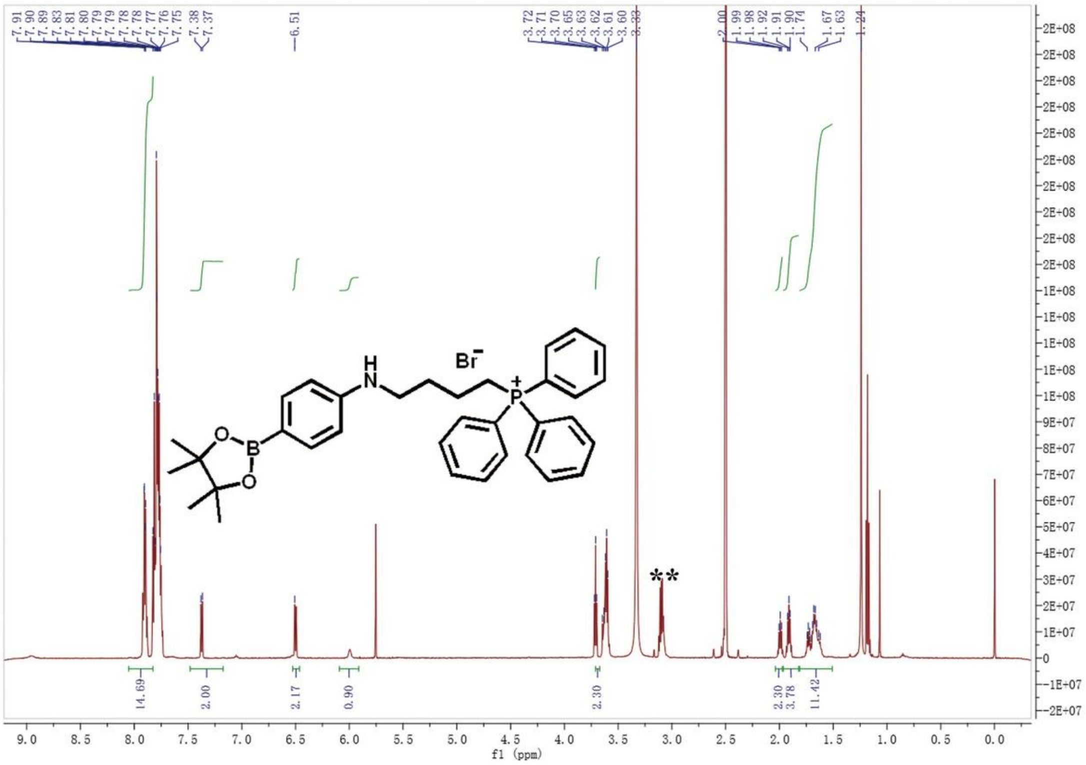

Supplementary Figure 39. The ^{1}HNMR spectrum of 4-Aminophenylboronic acid pinacol ester, **9**. (H_2O δ=3.3; DMSO-d_6 δ=2.5; the peak labeled with * was owing to dichloromethane; the peak labeled with ** was owing to methanol).

Synthesis of 5-fluoro-1-propargyl-uracil (13)

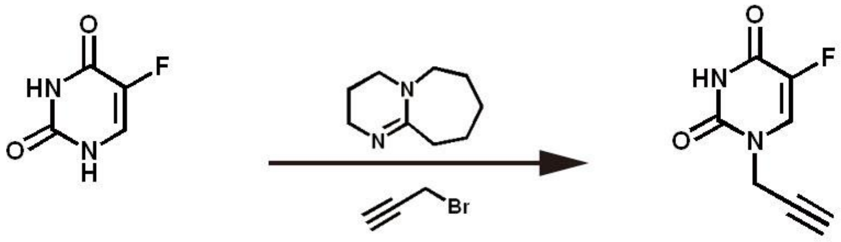

14 **13**

5-fluorouracil, **12**, (200 mg, 1.54 mmol) and 1,8-diazabicyclo[5.4.0]undec-7-ene (269 ml, 1.80 mmol) were dissolved in dry DMF (2 ml) under N_2 atmosphere and cooled to 4 °C. propargyl bromide (1.54 mmol) were dissolved in dry DMF (0.5 ml). The solution was added dropwise to the mixture and the resulting mixture stirred at room temperature overnight. Solvents were then removed under reduced pressure and the crude, **13**, purified via flash chromatography (3% MeOH in DCM).

^{1}H NMR (600 MHz, DMSO) δ 11.91 (d, *J* = 3.9 Hz, 1H), 8.13 (d, *J* = 6.6 Hz, 1H), 4.46 (d, *J* = 2.5 Hz, 2H), 3.44 (s, 1H).

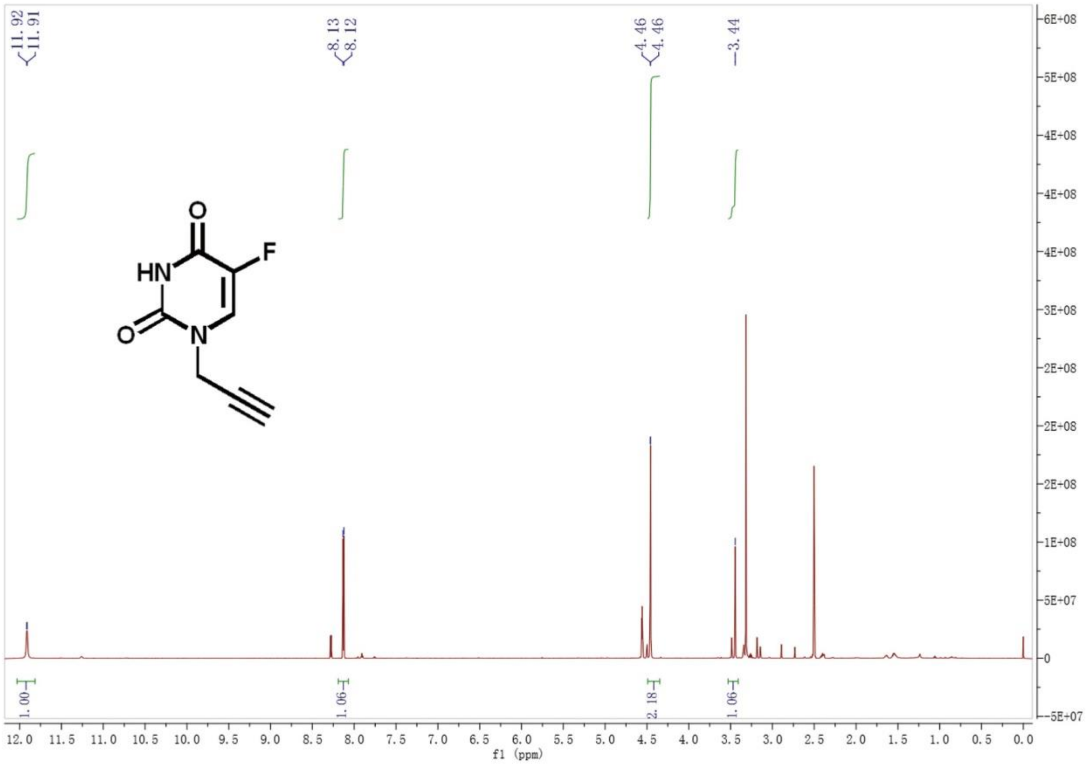

Supplementary Figure 40. The ^{1}HNMR spectrum of 5-fluoro-1-propargyl-uracil, **13**. (H_2O δ=3.3; DMSO-d_6 δ=2.5).

Supplementary References

1. Zhang, K. *et al.* Facile Large-Scale Synthesis of Monodisperse Mesoporous Silica Nanospheres with Tunable Pore Structure. *J. Am. Chem. Soc.* **135**, 2427-2430 (2013).
2. Lin, Y.H., Li, Z.H., Chen, Z.W., Ren, J.S. & Qu, X.G. Mesoporous silica-encapsulated gold nanoparticles as artificial enzymes for self-activated cascade catalysis. *Biomaterials* **34**, 2600-2610 (2013).
3. Ferris, D.P. *et al.* Light-Operated Mechanized Nanoparticles. *J. Am. Chem. Soc.* **131**, 1686-1688 (2009).
4. Gong, Y.H. *et al.* Photoresponsive smart template for reversible cell micropatterning. *J. Mat. Chem. B* **1**, 2013-2017 (2013).
5. Wang, J. *et al.* Chemical Remodeling of Cell-Surface Sialic Acids through a Palladium-Triggered Bioorthogonal Elimination Reaction. *Angew. Chem. Int. Edit.* **54**, 5364-5368 (2015).
6. Unciti-Broceta, A., Yusop, M.R., Richardson, P.R., Walton, J.G.A. & Bradley, M. A fluorescein-derived anthocyanidin-inspired pH sensor. *Tetrahedron Lett.* **50**, 3713-3715 (2009).
7. Yusop, R.M., Unciti-Broceta, A., Johansson, E.M.V., Sanchez-Martin, R.M. & Bradley, M. Palladium-mediated intracellular chemistry. *Nat. Chem.* **3**, 239-243 (2011).
